# 2-Oxoadenosine induces cytotoxicity through intracellular accumulation of 2-oxo-ATP and depletion of ATP but not via the p38 MAPK pathway

**DOI:** 10.1038/s41598-017-06636-8

**Published:** 2017-07-26

**Authors:** Shinji Asada, Eiko Ohta, Yoriko Akimoto, Nona Abolhassani, Daisuke Tsuchimoto, Yusaku Nakabeppu

**Affiliations:** 1 Division of Neurofunctional Genomics, Department of Immunobiology and Neuroscience, Medical Institute of Bioregulation, Kyushu University, Fukuoka, 812-8582 Japan; 2 Department of Medicine and Clinical Science, Graduate School of Medical Sciences, Kyushu University, Fukuoka, 812-8582 Japan

## Abstract

2-Oxoadenosine (2-oxo-Ado), an oxidized form of adenosine, is cytotoxic and induces growth arrest and cell death, which has potential as an anti-cancer drug. However, it is not well understood how 2-oxo-Ado exerts its cytotoxicity. We examined the effects of 2-oxo-Ado on non-tumour cells, namely immortalized mouse embryonic fibroblast lines, and investigated mechanisms by which 2-oxo-Ado exerts its cytotoxicity. We found that cell death induced by 2-oxo-Ado is classical caspase-dependent apoptosis, and requires its sequential intracellular phosphorylation catalysed by adenosine kinase (ADK) and adenylate kinase 2, resulting in intracellular accumulation of 2-oxo-ATP accompanied by accumulation of 2-oxo-Ado in RNA and depletion of ATP. Moreover, we showed that overexpression of MTH1, an oxidized purine nucleoside triphosphatase, prevents 2-oxo-Ado-induced cytotoxicity accompanied by suppression of accumulation of both intracellular 2-oxo-ATP and 2-oxo-Ado in RNA and recovery of ATP levels. We also found that 2-oxo-Ado activates the p38 MAPK pathway. However, siRNAs against *Mkk3* and *Mkk6*, or treatment with several p38 MAPK inhibitors, except SB203580, did not prevent the cytotoxicity. SB203580 prevented intracellular phosphorylation of 2-oxo-Ado to 2-oxo-AMP, and an *in vitro* ADK assay revealed that SB203580 directly inhibits ADK activity, suggesting that some of the effects of SB203580 may depend on ADK inhibition.

## Introduction

1,2-Dihydro-2-oxoadenosine (2-oxoadenosine; 2-oxo-Ado), an oxidized form of adenosine and also known as 2-hydroxyadenosine, isoguanosine or crotonoside, has been reported as a naturally generated nucleoside analogue in *Croton tiglium* ^1^ and *Diaulula sandiegensis* ^2^. 2-Oxo-Ado has not been identified in mammals, but some biological activities have been shown in mammals, such as effects on muscle contraction^3^ and cAMP accumulation^4^. Moreover, 2-oxo-Ado has an anti-tumour activity, namely toxicity that causes growth arrest and cell death of tumour-derived cell lines and suppression of tumorigenesis in a xenograft mouse model^5^. However, the mechanisms by which 2-oxo-Ado causes such cytotoxicity have not been elucidated.

It has been reported that 2-chloroadenosine (2-Cl-Ado) has an anti-tumour activity that induces apoptosis through its intracellular phosphorylation to 2-Cl-ATP, initiated by adenosine kinase (ADK)^6, 7^. Moreover, it has been shown that 8-chloroadenosine (8-Cl-Ado), which is also phosphorylated to the triphosphate form (8-Cl-ATP), induces cell death and activates the p38 mitogen-activated protein kinase (MAPK) pathway^8, 9^. Many studies have reported that growth arrest and cell death can be induced via the p38 MAPK pathway^10, 11^.

It is thus reasonable to assume that 2-oxo-Ado also induces cell death through its intracellular phosphorylation to 2-oxo-ATP, and its activation of the p38 MAPK pathway. We have shown that 2-oxo-ATP is efficiently hydrolysed to 2-oxo-AMP and pyrophosphate by an oxidized purine nucleoside triphosphatase, MTH1^12^, thereby raising the issue of whether the cytotoxicity caused by 2-oxo-Ado can be supressed by MTH1.

In the present study, we first confirmed that 2-oxo-Ado cytotoxicity against mouse embryonic fibroblast (MEF) lines is dependent on ADK and adenylate kinase 2 (AK2). We also found accumulation of 2-oxo-ATP with significant depletion of ATP in the cellular nucleotide pool. Over-expression of human MTH1 in an *Mth1*-knockout MEF line efficiently supressed 2-oxo-Ado-induced cytotoxicity with significant reduction of 2-oxo-ATP levels in the nucleotide pool. Moreover, we found that 2-oxo-Ado activates the p38 MAPK pathway through MKK3/MKK6 activation. However, such activation is dispensable for 2-oxo-Ado to exert its cytotoxicity.

## Results

### 2-Oxoadenosine induces cell death in immortalized mouse embryonic fibroblasts

**Figure 1:**
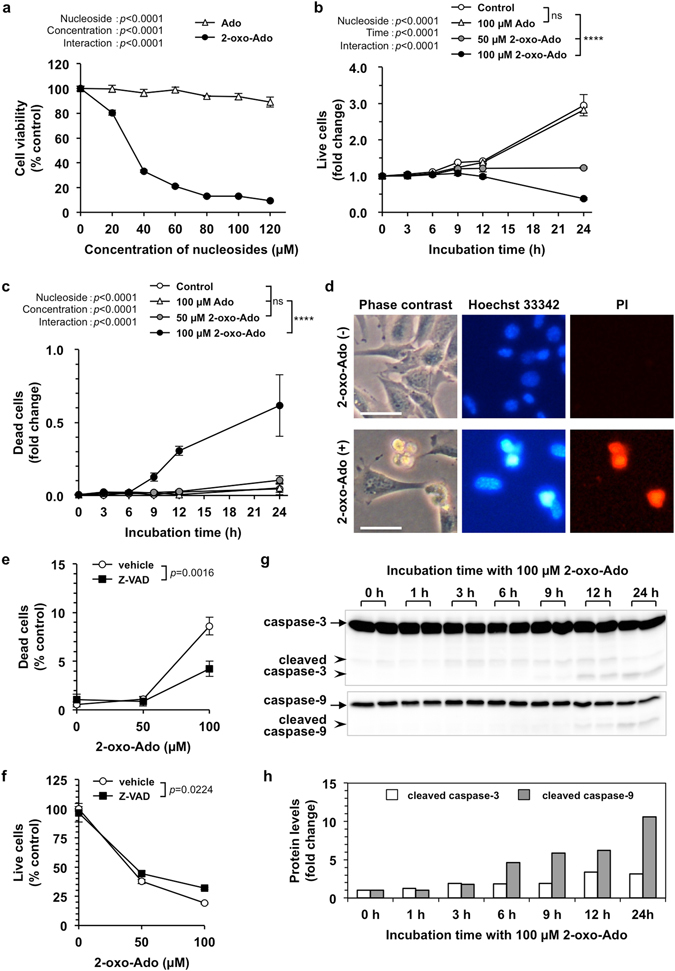
2-Oxo-Ado induces cell death in MEFs. (**a**) Cell viability of wild-type MEFs (T9 cells) was determined by WST-8 assays after incubation for 24 h in the presence of various concentrations of adenosine (Ado) or 2-oxoadenosine (2-oxo-Ado). Values (% control) of cell viability relative to that in the absence of nucleosides are shown as the mean ± SD of three experiments. (**b**,**c**) T9 cells were incubated in the presence of 100 µM Ado or 2-oxo-Ado for various times, and then the numbers of live and dead cells were determined by the trypan blue exclusion. Control, no additional nucleosides. Relative values of live (**b**) and dead (**c**) cell numbers to the initial number of total cells at time 0 are shown as the mean ± SD of three experiments. (**d**) Morphological alteration of T9 cells after exposure to 2-oxo-Ado. T9 cells incubated with (bottom panels) or without (upper panels) 100 µM 2-oxo-Ado for 24 h were stained with Hoechst 33342 and propidium iodide (PI). Images obtained by phase contrast and fluorescence microscopy are shown. Scale bars, 50 µm. (**e**,**f**) Numbers of live and dead T9 cells were determined by trypan blue exclusion at 24 h after incubation in the presence of 2-oxo-Ado (0, 50, and 100 µM) with or without 30 µM Z-VAD(OMe)-FMK (Z-VAD), a pan-caspase inhibitor. Relative values (% of control) of dead (**e**) and live (**f**) cell numbers to the total cell number in the absence of 2-oxo-Ado and Z-VAD (control) are shown as the mean ± SD of three experiments. (**g**) Protein levels in T9 cells were determined by western blot analysis after incubation with 100 µM 2-oxo-Ado for various times. Eight micrograms of total protein was applied to each lane. (**h**) Quantification of protein levels in (**g**). Results are normalised against total protein levels determined by Ponceau S staining. Relative values (fold change) of protein levels to those at time 0 are shown as the mean of two experiments. (**a**–**c**,**e**,**f**) Results were tested with two-way ANOVA and *post hoc* Tukey’s HSD test. ns, not significant; ****p < 0.0001.

We used a spontaneously immortalized mouse cell line (T9) derived from MEFs to evaluate the effects of 2-oxo-Ado on non-tumour cells. First, we exposed T9 cells to various concentrations of 2-oxo-Ado and Ado (20–120 µM) for 24 h and then assessed their viability using a tetrazolium salt (WST-8) assay (Fig. 1a). 2-Oxo-Ado significantly decreased the viability of T9 cells to less than 10% of the control level, in a dose-dependent manner up to 80 µM, whereas Ado had little effect even at 120 µM.

Next, we performed trypan blue exclusion assays at various time points after incubation of T9 cells with or without 2-oxo-Ado (50 and 100 µM) or Ado (100 µM). The number of live and dead cells at each time point relative to the initial number of total cells is shown in Fig. 1b,c. The number of live cells doubled within 24 h with or without 100 µM Ado, indicating that 100 µM Ado had no toxic effect on T9 cells in this assay. Conversely, the number of live cells cultured in the presence of 50 µM 2-oxo-Ado did not increase within 24 h. Moreover, the number of live cells was significantly decreased to 37.5% of the initial number and the dead cell number was significantly increased after incubation for 24 h with 100 µM 2-oxo-Ado, but not 50 µM 2-oxo-Ado. These results indicate that 2-oxo-Ado induces growth arrest and cell death in a dose-dependent manner. Based on these results, we concluded that 2-oxo-Ado has toxic effects on non-tumour cells by inducing growth arrest and cell death.

In cultures of T9 cells exposed to 100 µM 2-oxo-Ado, nuclei of propidium iodide (PI)-positive dead cells were small and condensed (Fig. 1d). Z-VAD(OMe)-FMK, a pan-caspase inhibitor^13^, significantly suppressed cell death and increased cell survival (Fig. 1e,f), suggesting that cell death caused by 2-oxo-Ado is a caspase-dependent apoptosis. Indeed, exposure of T9 cells to 2-oxo-Ado induced cleavage of caspase-3 and -9 (Fig. 1g,h), both of which are essential to activate the classical mitochondrial apoptotic pathway^14^. We thus concluded that T9 MEF cells exposed to 100 µM 2-oxo-Ado undergo classical caspase-dependent apoptosis.

We also examined the effects of 2-oxo-Ado on normal human fibroblasts and tumour-derived cell lines (Supplementary Fig. S1). WI38 cells, normal diploid human fibroblasts derived from lung tissue, were resistant to 2-oxo-Ado up to 120 µM with a mild growth suppression at 80–120 µM. U2OS cells, a human osteosarcoma cell line, were also resistant to 2-oxo-Ado, but their growth was completely suppressed at 120 µM. In contrast, MOLT4 cells, a human acute lymphoblastic leukaemia cell line, were quite sensitive to 2-oxo-Ado, exhibiting apparent cell death at 80–120 µM. These results indicate that the cytotoxicity of 2-oxo-Ado is dependent on the cell type. Thus, we continued its characterization using MEFs in this study.

### Induction of MEF cell death requires intracellular phosphorylation of 2-oxoadenosine by adenosine kinase

**Figure 2:**
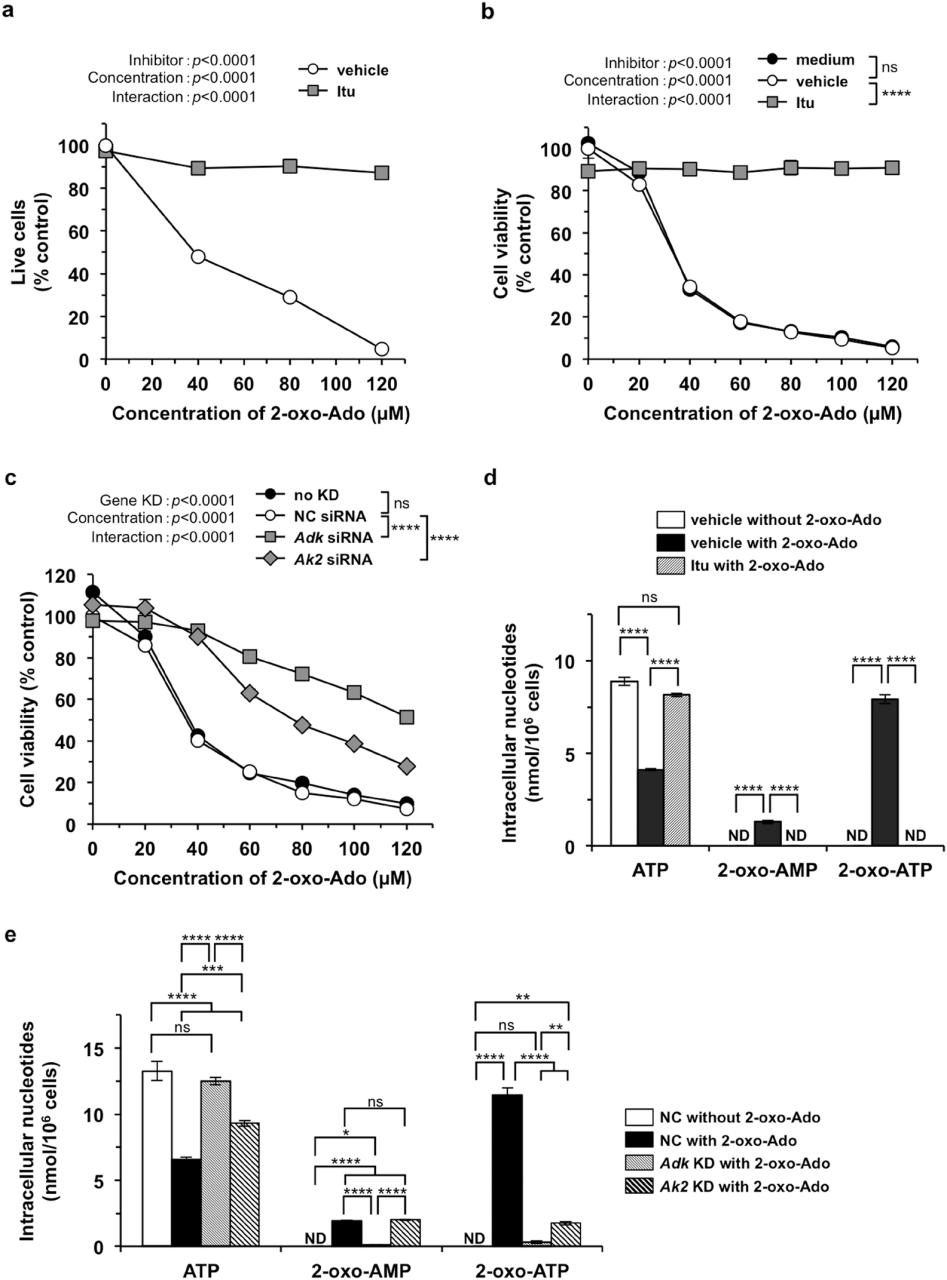
2-Oxo-Ado-induced cell death requires intracellular phosphorylation of 2-oxo-Ado initiated by adenosine kinase (Adk). (**a**) The number of live T9 cells was determined by trypan blue exclusion after incubation for 24 h in the presence of various concentrations of 2-oxo-Ado with or without 0.1 µM 5-Iodtubercidin (Itu), an Adk inhibitor. Vehicle control, 0.1% DMSO. Values (% control) of the number of live cells relative to that of control cells in the absence of 2-oxo-Ado are shown as the mean ± SD of three experiments. (**b**,**c**) Viability of T9 cells was determined by WST-8 assays after incubation for 24 h in the presence of various concentrations of 2-oxo-Ado. (**b**) Cells were incubated with 0.1 µM Itu, vehicle (0.1% DMSO), or without them (medium). (**c**) Cells were treated with target siRNA for gene-knockdown (KD) or negative control (NC) siRNA as the control at 48 h before commencement of 2-oxo-Ado treatment. Non-KD-treated cells (no KD) were also used. Values (% control) of cell viability relative to that of control cells in the absence of 2-oxo-Ado are shown as the mean ± SD of three experiments. (**d**,**e**) Intracellular nucleotide concentrations of T9 cells were determined by HPLC after incubation for 6 h in the presence or absence of 100 µM 2-oxo-Ado. Cells were treated with the vehicle (0.3% DMSO), 0.1 µM Itu (**d**), or siRNAs against *Adk* and *Ak2* (**e**). Results are shown as the mean ± SD of three experiments. ND; not detected. (**a**–**e**) Results were statistically analysed by two-way (**a**–**c**) or one-way (**d**,**e**) ANOVA and *post-hoc* Tukey’s HSD test among inhibitor conditions (**b**), different KD conditions (**c**) or each kind of nucleotide (**d**,**e**). ns, not significant; *p < 0.05; **p < 0.01; ***p < 0.001; and ****p < 0.0001.

To examine whether 2-oxo-Ado is required to be phosphorylated to 2-oxo-ATP by ADK to exert its effects, we exposed T9 cells to various concentrations of 2-oxo-Ado in the presence or absence of 0.1 µM 5-iodotubercidin (Itu), an inhibitor of ADK^15^, and then performed trypan blue exclusion assays (Fig. 2a). In the presence of 0.1 µM Itu, exposure to increasing concentrations of 2-oxo-Ado (40 to 120 µM) only slightly reduced the numbers of live cells, indicating that Itu efficiently suppressed the cytotoxicity of 2-oxo-Ado. As shown in Fig. 2b, WST-8 assays also revealed that Itu completely suppressed the severe reduction of cell viability caused by increasing concentrations of 2-oxo-Ado (20–120 µM), although Itu itself only slightly reduced cell viability and the vehicle had no effect on the cytotoxicity of 2-oxo-Ado.

We next evaluated the effects of small interfering RNA (siRNA)-mediated knockdown of *Adk* or adenylate kinase 2 (*Ak2*) on the cytotoxicity of 2-oxo-Ado. *Ak2* encodes one of the adenylate kinase isozymes responsible for the reversible transfer of phosphate groups among adenine nucleotides^16^ and possibly also on 2-oxoadenine nucleotides. T9 cells were pre-treated with siRNAs for 48 h (Supplementary Fig. S2), exposed to various concentrations of 2-oxo-Ado for 24 h, and then subjected to WST-8 assays (Fig. 2c). siRNA against *Adk* significantly suppressed the cytotoxicity of 2-oxo-Ado compared with the effect of negative control (NC) siRNA, although the suppression was slightly less efficient than that achieved by Itu. siRNA against *Ak2* also significantly suppressed the cytotoxicity of 2-oxo-Ado, but less efficiently than *Adk* siRNA, probably reflecting the presence of multiple isozymes such as AK1 or AK3.

Taken together, these data indicate that the cytotoxicity of 2-oxo-Ado requires intracellular phosphorylation of 2-oxo-Ado to 2-oxo-AMP, 2-oxo-ADP and 2-oxo-ATP. To confirm intracellular phosphorylation of 2-oxo-Ado, we extracted intracellular nucleotides from T9 cells after a 6 h of exposure to 100 µM 2-oxo-Ado and 0.1 µM Itu, and subjected them to quantitative high-performance liquid chromatography (HPLC) (Fig. 2d, Supplementary Fig. S3). In control T9 cells without exposure to 2-oxo-Ado, approximately 9 nmol ATP per 1 × 10^6^ cells was detected, but no 2-oxo-Ado, 2-oxo-AMP, 2-oxo-ADP, 2-oxo-ATP was detected. In the HPLC condition, AMP was not detected, while ADP was merged with an unidentified peak. Therefore, we could not measure the amount of ADP. After exposure to 100 µM 2-oxo-Ado, approximately 70–90 pmol 2-oxo-Ado, 1.3 nmol 2-oxo-AMP and 8 nmol 2-oxo-ATP per 1 × 10^6^ cells were detected, while ATP levels were decreased to 46% of the level detected in control T9 cells. The area of peaks containing ADP was not altered after exposure to 2-oxo-Ado, suggesting that the ADP level was unaffected by exposure to 2-oxo-Ado. After exposure to 2-oxo-Ado in the presence of 0.1 µM Itu, only ATP (approximately 8 nmol per 1 × 10^6^ cells) was detected, and 2-oxo-AMP, 2-oxo-ADP and 2-oxo-ATP were not detected. Similar to the ADK inhibitor, siRNA-mediated knockdown of *Adk* or *Ak2* inhibited both intracellular accumulation of 2-oxo-ATP and the reduction of ATP (Fig. 2e). Neither inhibition of ADK nor knockdown of *Adk* or *Ak2* altered intracellular concentrations of 2-oxo-Ado (Supplementary Fig. S3). These results confirmed that 2-oxo-Ado was indeed phosphorylated to 2-oxo-ATP in T9 cells, which was dependent on both ADK and AK2.

**Figure 3:**
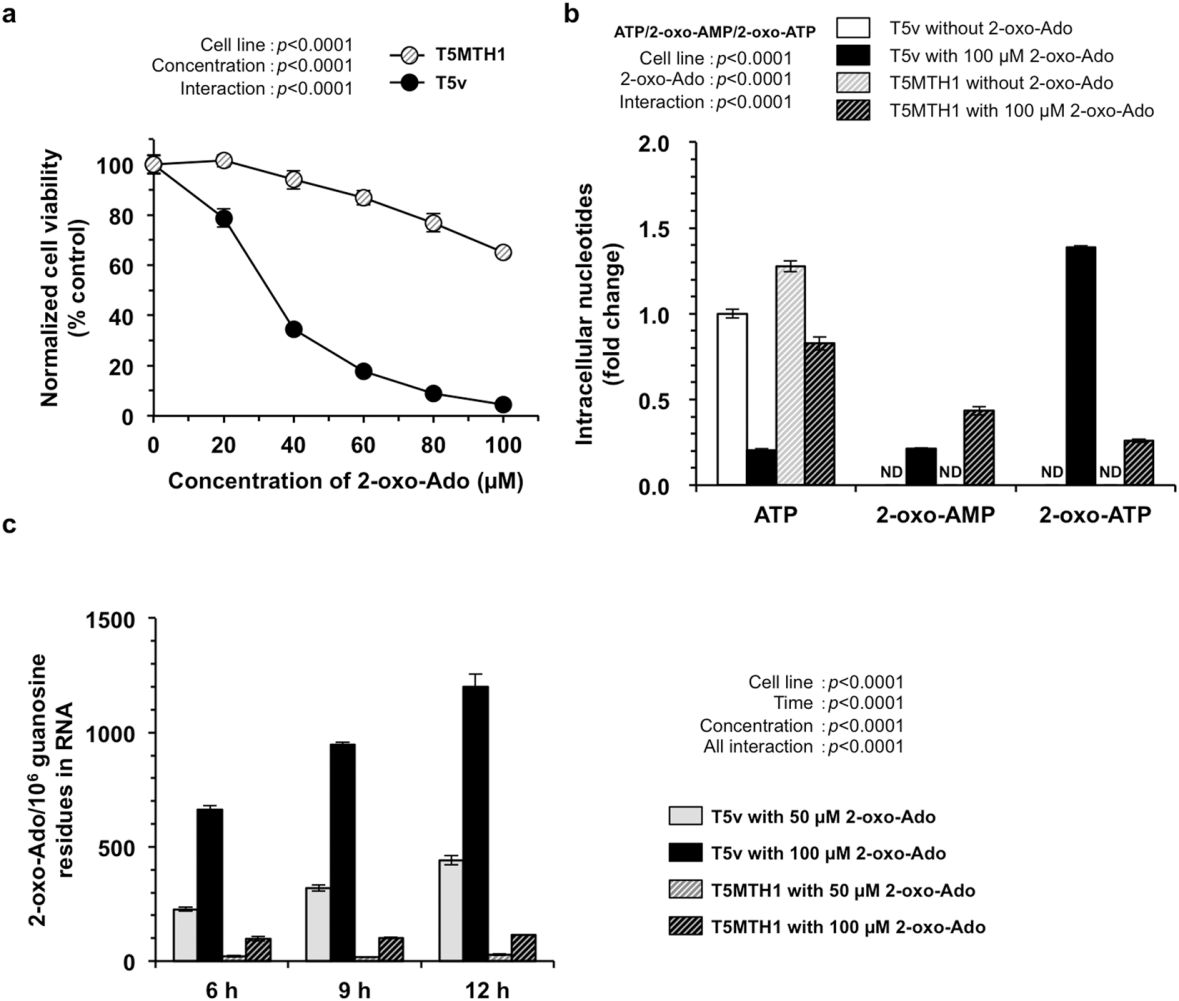
Human MTH1 prevents 2-oxo-Ado-induced cell death. (**a**) Cell viability of mouse MTH1-null MEFs (T5v cells) and mouse MTH1-null/human MTH1-expressing MEFs (T5MTH1 cells) was determined by WST-8 assays after incubation for 24 h in the presence of various concentrations of 2-oxo-Ado. Values (% control) of cell viability relative to that in the absence of 2-oxo-Ado for each cell line are shown as the mean ± SD of three experiments. Results were statistically analysed by two-way ANOVA. (**b**) Intracellular nucleotide concentrations of T5v and T5MTH1 cells were determined by HPLC after incubation for 6 h in the presence or absence of 100 µM 2-oxo-Ado. Values (fold change) of indicated nucleotides relative to ATP in T5v cells in the absence of 2-oxo-Ado are shown as the mean ± SD of three experiments. The concentration of ATP in T5v cells in the absence of 2-oxo-Ado was 11.6 nmol/1 × 10^6^ cells and that in T5MTH1 cells was 14.9 nmol/1 × 10^6^ cells. Results were statistically analysed by two-way ANOVA for ATP, 2-oxo-AMP, 2-oxo-ATP, separately. (**c**) 2-Oxo-Ado residues relative to 1 × 10^6^ guanosine residues in RNA of T5v and T5MTH1 cells was determined by LC-MS/MS. RNAs were collected after incubation with the indicated concentrations of 2-oxo-Ado for various times. Results are shown as the mean ± SD of three experiments. Results were statistically analysed by three-way ANOVA.

Because the cytotoxicity of 2-oxo-Ado was partly dependent on AK2 without detectable accumulation of 2-oxo-ADP, intracellular 2-oxo-ATP is most likely to be responsible for the cytotoxicity of 2-oxo-Ado. 2-Oxo-ATP is known to be efficiently hydrolysed by MTH1^12^. Therefore, we assumed that increased levels of MTH1 may decrease intracellular levels of 2-oxo-ATP, thus suppressing 2-oxo-Ado cytotoxicity. To examine this possibility, we used two cell lines, T5v and T5MTH1. Both are derived from a MEF line (T5) established from an *Mth1*-knockout mouse embryo^17^. T5v cells are deficient for MTH1 and carry an empty vector, while T5MTH1 cells express high levels of human MTH1 (hMTH1) protein (630 ng/mg total protein)^17^. As shown in Fig. 3a, T5v cells exhibited essentially the same sensitivity to increased concentrations of 2-oxo-Ado as T9 cells, probably because the endogenous level of mouse MTH1 in T9 cells is very low^17^. Conversely, T5MTH1 cells exhibited a significant increase in resistance to 2-oxo-Ado cytotoxicity.

To confirm the intracellular level of 2-oxo-ATP, we extracted intracellular nucleotides from T5v and T5MTH1 cells after exposure to 100 µM 2-oxo-Ado for 6 h, and then performed quantitative HPLC analysis (Fig. 3b). In T5v cells exposed to 2-oxo-Ado, 16.1 nmol 2-oxo-ATP per 1 × 10^6^ cells was detected, which was 1.38-fold higher than the level of ATP in control T5v cells without exposure to 2-oxo-Ado. Furthermore, T5v cells exposed to 2-oxo-Ado contained approximately 20% of the ATP seen in control T5v cells. However, in T5MTH1 cells exposed to 2-oxo-Ado, 3.0 nmol 2-oxo-ATP and 9.6 nmol ATP per 1 × 10^6^ cells were detected with a 2-fold increase in the 2-oxo-AMP level compared with T5v cells exposed to 2-oxo-Ado.

We next isolated RNA from T5v and T5MTH1 cells after exposure to 50 or 100 µM 2-oxo-Ado for 6–12 h, and determined the levels of 2-oxo-Ado in RNA by liquid chromatography-tandem mass spectrometry (LC-MS/MS) analysis (Fig. 3c). In T5v cells, 2-oxo-Ado levels in RNA were increased after exposure to 2-oxo-Ado in time- and dose-dependent manners. About 1200 residues of 2-oxo-Ado per 1 × 10^6^ guanosine residues in RNA were detected in T5v cells after 12 h of exposure to 100 µM 2-oxo-Ado, while only 114 residues of 2-oxo-Ado per 1 × 10^6^ guanosine residues in RNA (about 10% of that in T5v cells) were detected in T5MTH1 cells after the exposure. It is noteworthy that basal levels of 2-oxo-Ado in untreated T5v and T5MTH1 cells were under the limit of detection (one residue of 2-oxo-Ado per 1 × 10^7^ guanosine residues in RNA).

We thus concluded that the cytotoxicity of 2-oxo-Ado is dependent on intracellular formation of 2-oxo-ATP mediated by ADK and AK2, which results in remarkable accumulation of 2-oxo-Ado in RNA and depletion of ATP. In addition, hMTH1 prevents accumulation of 2-oxo-Ado in RNA as well as accumulation of 2-oxo-ATP and depletion of ATP, and thus suppresses the cytotoxicity.

### 2-Oxoadenosine activates the p38 MAPK pathway which is dispensable for its cytotoxicity

**Figure 4:**
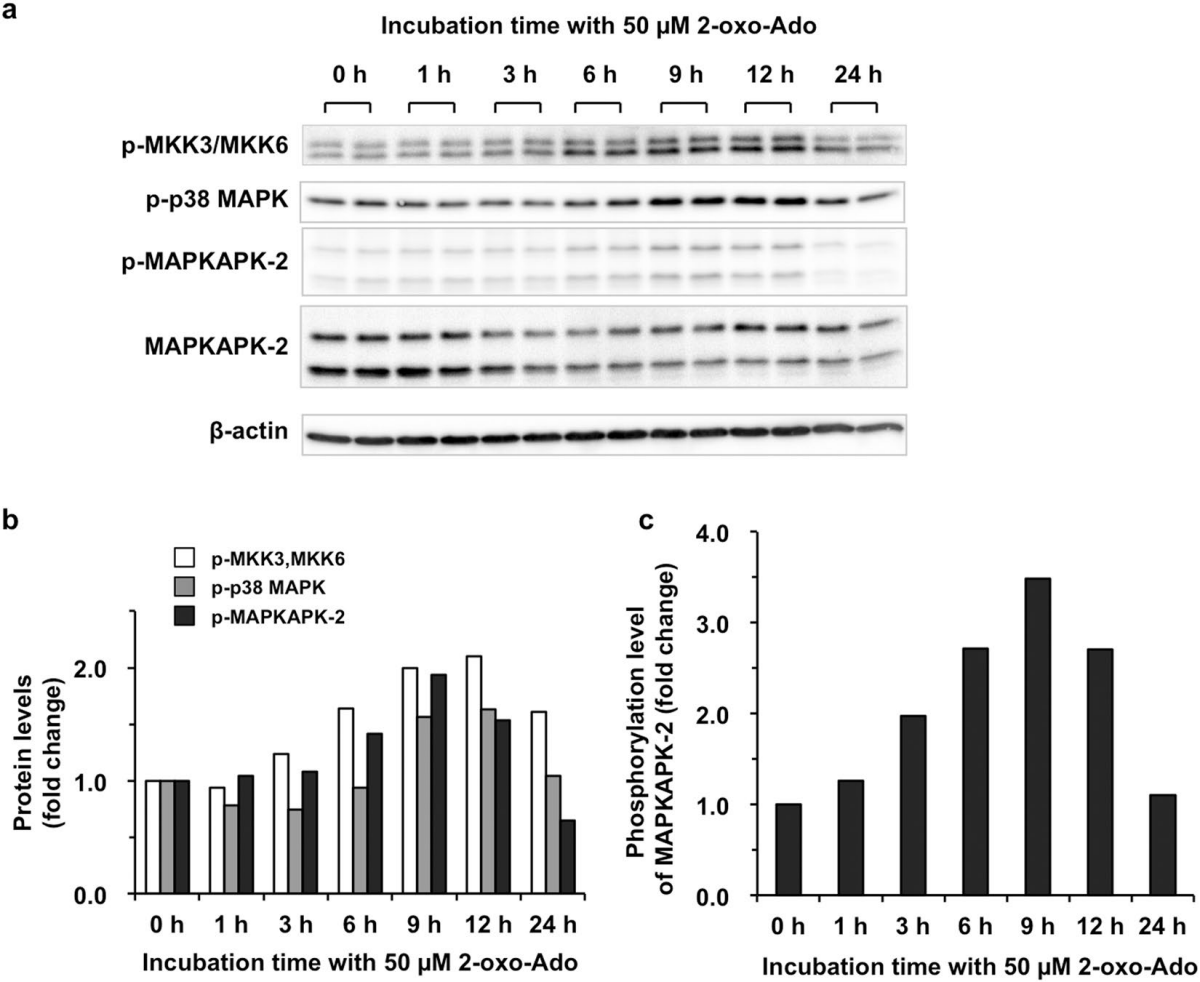
2-Oxo-Ado activates the p38 MAPK pathway. (**a**) Protein levels of T9 cells were determined by western blot analysis after incubation with 50 µM 2-oxo-Ado for various times. Ten micrograms of total protein was applied to each lane. p-; phosphorylated. (**b**,**c**) Quantification of protein levels in (**a**). Results are normalized against β-actin in (**b**), and relative values (fold change) of protein (**b**) or phosphorylation levels (**c**) to those at time 0 are shown as the mean of two experiments.

We next examined whether 2-oxo-Ado activates p38 MAPKs (α, β, γ, and δ) prior to cell death. We evaluated phosphorylation of p38 MAPK kinases (p38 MAPKKs: MKK3^18^ and MKK6^19^), p38 MAPKs and their substrate (MAPKAPK-2)^20, 21^ in T9 cells after exposure to 50 µM 2-oxo-Ado (Fig. 4). Increased phosphorylation of these proteins was observed as early as 3 h after exposure, reaching maximum levels at 9–12 h after exposure, indicating that 2-oxo-Ado activates the p38 MAPK signalling pathway.

**Figure 5:**
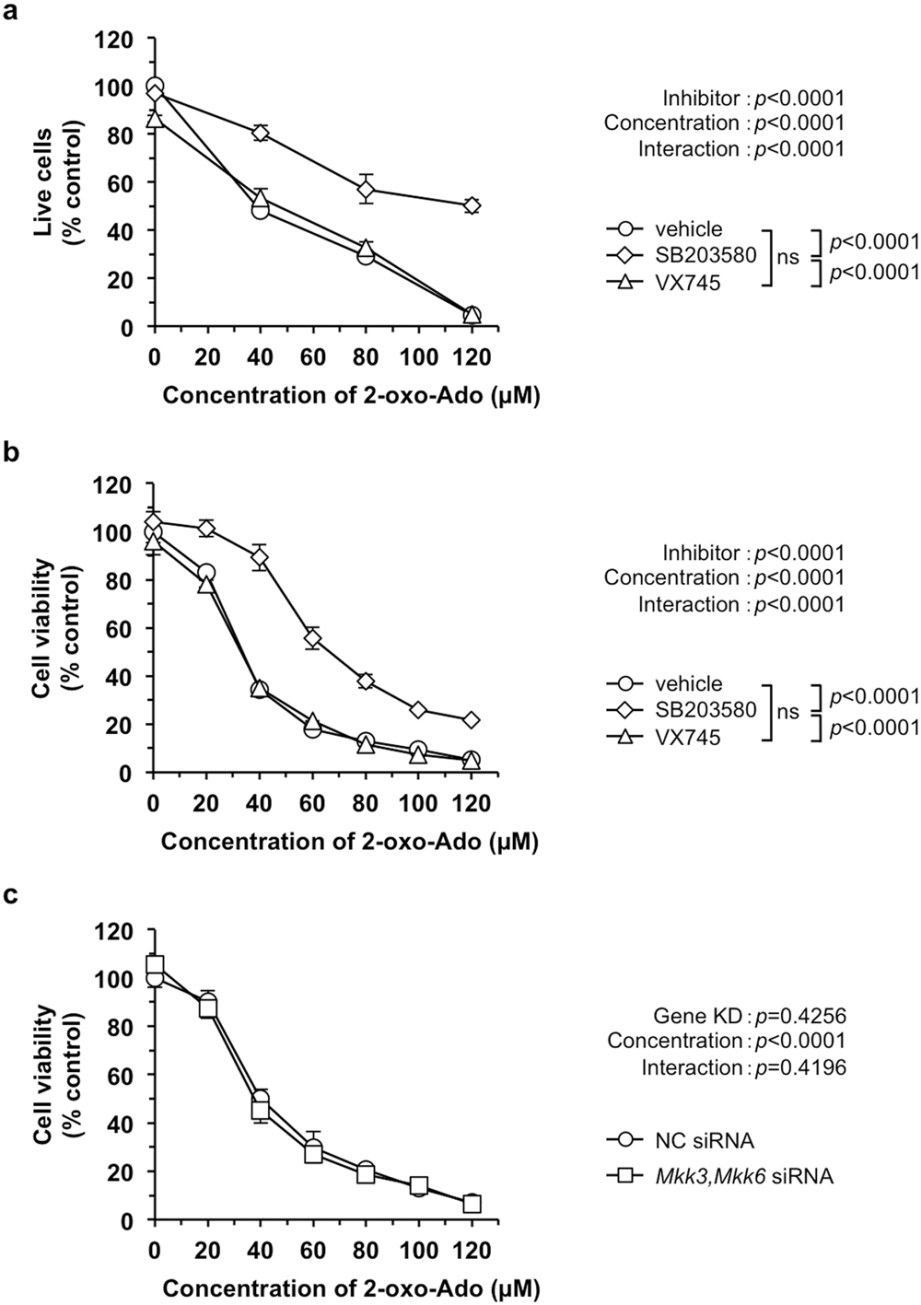
SB203580 blocks 2-oxo-Ado-induced cell death but not via inhibition of the p38 MAPK pathway. (**a**,**b**) The number of live T9 cells was determined by trypan blue exclusion (**a**), and cell viability of T9 cells was determined by WST-8 assays (**b**). Cells were incubated in the presence of various concentrations of 2-oxo-Ado with or without the indicated inhibitors for 24 h. SB203580 and VX745, 10 µM. Vehicle control, 0.1% DMSO. Values (% control) of the number of live cells relative to that of the control in the absence of 2-oxo-Ado are shown as the mean ± SD of three experiments. (**c**) Cell viability of T9 cells was determined by WST-8 assays. Cells were treated with target siRNAs or NC siRNA as the control for 48 h and then incubated with various concentrations of 2-oxo-Ado for 24 h. Values (% control) of cell viability relative to that of the control in the absence of 2-oxo-Ado are shown as the mean ± SD of three experiments. Results were statistically analysed by two-way ANOVA (**a**–**c**) and *post-hoc* Tukey’s HSD test among different inhibitor conditions (**a**,**b**).

To examine whether p38 MAPK activation is essential for 2-oxo-Ado-induced cell death, we applied four different types of p38 MAPK inhibitors, SB203580, SB202190, VX745 and BIRB796, to T9 cells with or without exposure to 2-oxo-Ado. As shown in Supplementary Fig. S4, while the increased levels of p38 MAPK phosphorylation after exposure to 2-oxo-Ado for 9 h were not altered in the presence of any inhibitor, phosphorylation levels of MAPKAPK-2 were markedly decreased in the presence of all four inhibitors to below the basal levels observed without exposure to 2-oxo-Ado. These results confirmed that all four inhibitors efficiently inhibited p38 MAPK activity, but not its phosphorylation, thus preventing activation of p38 MAPK downstream signalling in T9 cells exposed to 2-oxo-Ado. However, among these inhibitors, only SB203580 significantly suppressed the cytotoxicity of 2-oxo-Ado, as revealed by trypan blue exclusion and WST-8 assays (Fig. 5a,b, Supplementary Fig. S5). VX745 and BIRB796 had no effect on the viability of T9 cells exposed to increasing concentrations of 2-oxo-Ado (20–120 µM). While SB202190 slightly improved cell viability even without exposure to 2-oxo-Ado, there was no significant interaction between the two factors in two-way analysis of variance (ANOVA), indicating that SB202190 did not suppress the cytotoxicity of 2-oxo-Ado (Supplementary Fig. S5).

To clarify whether p38 MAPK activation is essential for 2-oxo-Ado-induced cell death, we next treated T9 cells with siRNAs against *Mkk3* and *Mkk6*, with or without exposure to 2-oxo-Ado (Supplementary Fig. S6). siRNA against *Mkk3*, which encodes a major p38 MAPKK in T9 cells, increased expression of *Mkk6* mRNA^22^. Thus, we simultaneously treated T9 cells with two siRNAs against *Mkk3* and *Mkk6*, which achieved efficient knockdown of both *Mkk3* and *Mkk6* mRNAs. When T9 cells were pre-treated for 48 h with siRNAs against *Mkk3* and *Mkk6*, phosphorylation levels of p38 MAPKs and MAPKAPK-2 induced by a 9 h of exposure to 50 µM 2-oxo-Ado were significantly reduced, even below the basal levels seen in T9 cells treated with NC siRNA in the absence of 2-oxo-Ado. This result demonstrated functional silencing of p38 MAPK signalling. We then examined the effects of *Mkk3* and *Mkk6* knockdown on the cytotoxicity of 2-oxo-Ado (Fig. 5c) and found no suppression of cytotoxicity caused by increased concentrations of 2-oxo-Ado (20–120 µM).

These results clearly showed that exposure of T9 cells to 2-oxo-Ado activates the p38 MAPK signalling pathway. However, such activation is not necessary for T9 cells to undergo 2-oxo-Ado-induced cytotoxicity. It was also noteworthy that SB203580 suppressed 2-oxo-Ado-induced cytotoxicity and that this suppression must be mediated by an unknown function of this compound and not from inhibition of p38 MAPK activity.

### SB203580 inhibits adenosine kinase activity and suppresses 2-oxo-Ado-cytotoxicity

**Figure 6:**
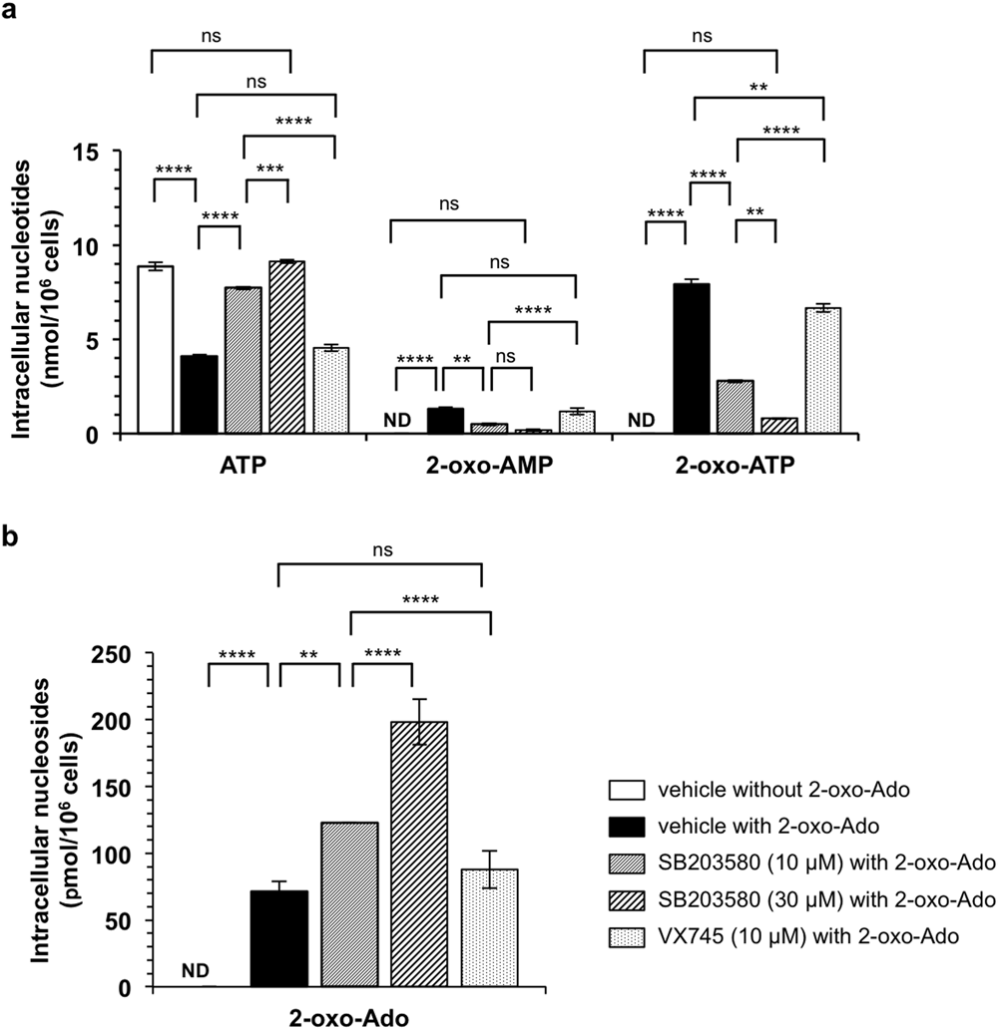
SB203580 inhibits phosphorylation of 2-oxo-Ado to 2-oxo-AMP but not via inhibition of the p38 MAPK pathway. (**a**,**b**) Intracellular concentrations of nucleotides (**a**) or nucleosides (**b**) of T9 cells were determined by HPLC after incubation for 6 h with or without 100 µM 2-oxo-Ado and the indicated inhibitors. Vehicle control, 0.3% DMSO. Results are shown as the mean ± SD of three experiments. ND; not detected. Results were statistically analysed by one-way ANOVA and *post-hoc* Tukey’s HSD test for each kind of nucleotides or nucleoside. ns, not significant; **p < 0.01; ***p < 0.001; and ****p < 0.0001.

Suppression of 2-oxo-Ado-cytotoxicity by SB203580 independently of p38 MAPK inhibition may provide a clue to understand the mechanism by which 2-oxo-Ado exerts its cytotoxicity. Because 2-oxo-Ado must be converted to 2-oxo-ATP or cause depletion of ATP to induce cell death, we first evaluated whether SB203580 alters the levels of these nucleotides in T9 cells exposed to 100 µM 2-oxo-Ado for 6 h (Fig. 6a). When cells were incubated for 6 h in the presence of 100 µM 2-oxo-Ado with or without SB203580 (10 and 30 µM), SB203580 significantly suppressed both the reduction of ATP levels and the accumulation of 2-oxo-AMP and 2-oxo-ATP in a dose-dependent manner. While VX745 also significantly suppressed accumulation of 2-oxo-ATP compared with T9 cells exposed to 100 µM 2-oxo-Ado in the absence of the p38 MAPK inhibitor, the level of 2-oxo-ATP was significantly higher than that in T9 cells exposed to 2-oxo-Ado in the presence of SB203580 (10 and 30 µM). The reduction of ATP levels and the accumulation of 2-oxo-AMP in T9 cells induced by 2-oxo-Ado was not altered by VX745. Under the same conditions, intracellular 2-oxo-Ado levels were significantly increased in the presence of SB203580 in a dose-dependent manner, but not in the presence of VX745 (Fig. 6b). These results suggest that SB203580 inhibits conversion of 2-oxo-Ado to 2-oxo-AMP, which is catalysed by ADK.

We next investigated the effect of SB203580 on other ADK-dependent signalling reaction, namely activation of AMP-activated protein kinase (AMPK) by 5-aminoimidazole-4-carboxamide-1-β-d-ribofuranoside (AICAR)^23^ (Supplementary Fig. S7). SB203580 and, to a lesser extent, SB202190 and BIRB796 significantly inhibited phosphorylation of AMPK in the presence of AICAR, whereas VX745 did not inhibit AMPK phosphorylation.

**Figure 7:**
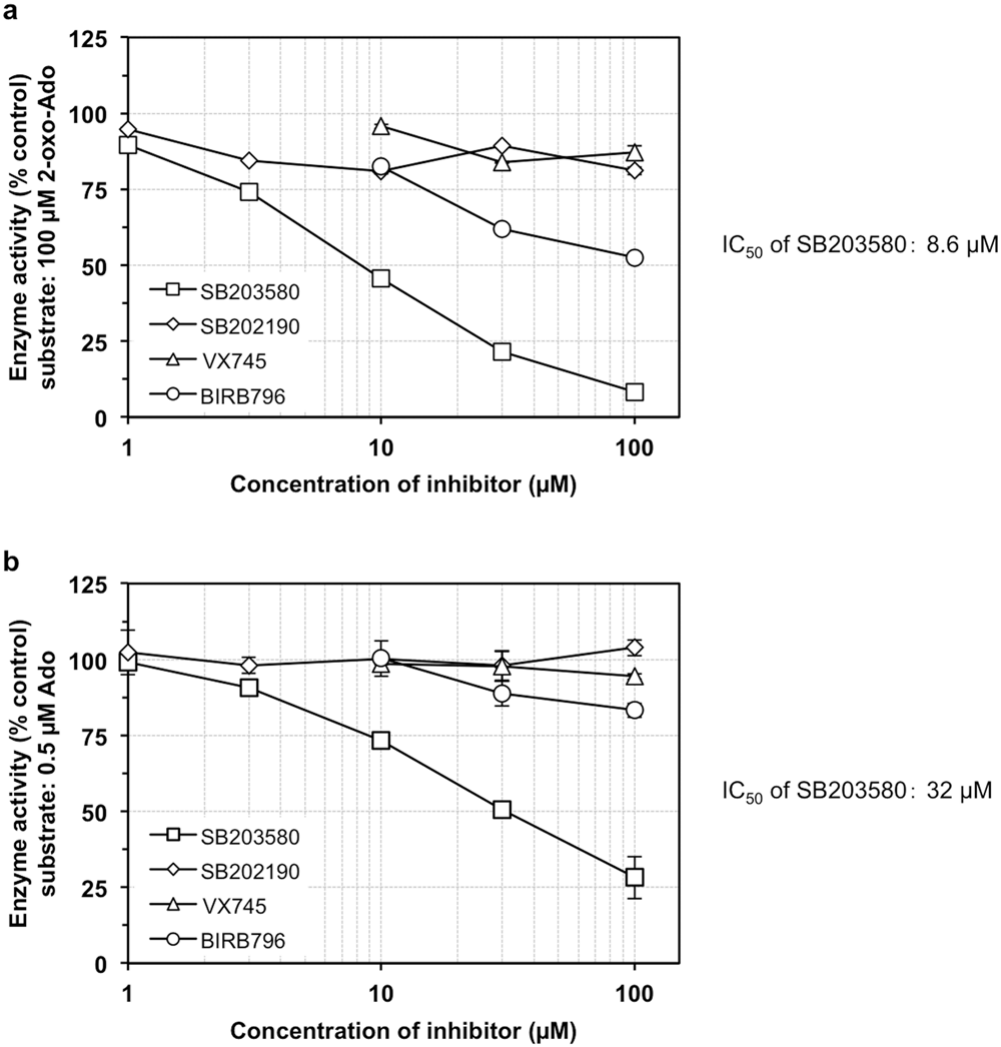
SB203580 directly inhibits adenosine kinase. (**a**,**b**) Recombinant human ADK, ATP and nucleosides 2-oxo-Ado (**a**) or Ado (**b**) were reacted in the presence of various concentrations of inhibitors. Enzyme activity was determined by the amount of monophosphate products, 2-oxo-AMP (**a**) or AMP (**b**). Product quantities were determined by HPLC. Values (% control) of enzyme activity relative to that in the absence of inhibitors are shown as the mean ± SD of three experiments. IC_50_ was calculated graphically.

These results suggest that SB203580 directly inhibits or indirectly alters ADK activity. To examine the direct effect of SB203580 on ADK activity, we performed *in vitro* enzyme assays with recombinant human ADK (rhADK) (Fig. 7). When 100 µM of 2-oxo-Ado was incubated in the presence of rhADK and ATP, 2-oxo-AMP-formation was significantly inhibited by SB203580 (IC_50_ = 8.6 µM) and to a lesser extent by BIRB796 (IC_50_ ≥ 100 µM) in a dose-dependent manner, but VX745 had little inhibitory effect on ADK up to 100 µM. When 0.5 µM Ado was incubated in the presence of rhADK and ATP, only SB203580 exhibited significant inhibition of AMP formation (IC_50_ = 32 µM). As shown in Supplementary Fig. S8, essentially the same results were obtained with other adenosine analogues, AICAR and 2-chloroadenosine (2-Cl-Ado), as those obtained with 2-oxo-Ado. We thus concluded that SB203580 directly inhibits ADK activity.

2-Cl-Ado is also known to induce cell death that is dependent on ADK and 2-Cl-ATP formation^6^. Therefore, we examined whether SB203580 suppresses its cytotoxicity in T5v and T5MTH1 cells (Supplementary Fig. S9). T5v and T5MTH1 cells exhibited essentially the same sensitivity to increased concentrations of 2-Cl-Ado (20–120 µM), indicating that MTH1 may not hydrolyse 2-Cl-ATP. Itu at 0.1 µM efficiently suppressed 2-Cl-Ado cytotoxicity, confirming that ADK is required for toxicity in MEFs, while 10 µM SB203580, but not VX745, also significantly suppressed cytotoxicity although less efficiently than Itu.

We thus demonstrated that 2-oxo-Ado causes growth arrest and apoptosis dependent on ADK, AK2 and the formation of 2-oxo-ATP (triphosphate form) similarly to various modified adenosines, such as 8-Cl-Ado and 2-Cl-Ado, which are potent anti-cancer drugs.

## Discussion

In the present study, we showed that exposure of immortalized MEFs to 2-oxo-Ado induces growth arrest and classical caspase-dependent apoptosis, accompanied by accumulation of intracellular 2-oxo-ATP, resulting in accumulation of 2-oxo-Ado in cellular RNA, and depletion of ATP, both of which are dependent on ADK and AK2. Overexpression of human MTH1, an oxidized purine nucleoside triphosphatase, efficiently suppressed 2-oxo-Ado-induced cytotoxicity with significantly reduced accumulation of both intracellular 2-oxo-ATP and 2-oxo-Ado in cellular RNA, as well as recovery of ATP levels. Furthermore, 2-oxo-Ado activated the p38 MAPK pathway dependent on MKK3/MKK6. However, such activation was not necessary for 2-oxo-Ado to exert its cytotoxicity.

Exposure of T9 cells to 2-oxo-Ado caused intracellular accumulation of 2-oxo-ATP to a level equivalent to the normal level of intracellular ATP and halved the ATP level as early as 6 h after exposure. Both of these effects were almost completely prevented by Itu, an ADK inhibitor (Fig. 2). The cytotoxicity induced by 2-oxo-Ado was almost completely suppressed by Itu, and partially by knockdown of *Adk* or *Ak2*. These results indicate that 2-oxo-Ado-induced cytotoxicity is mainly dependent on intracellular phosphorylation of 2-oxo-Ado but not on extracellular adenosine receptors, which are known to mediate various effects of adenosine analogues^24, 25^. However, phosphorylation of 2-oxo-AMP and 2-oxo-ADP by adenylate kinase is reversible depending on their concentration. Thus we could not explain why the 2-oxo-ATP level was so highly increased without accumulation of 2-oxo-ADP. Considering that the intracellular level of accumulated 2-oxo-ATP was equivalent to the normal level of intracellular ATP, 2-oxo-ATP may be actively generated by the same mechanism as ATP, namely oxidative phosphorylation in mitochondria, and it has been reported that 8-Cl-ADP is phosphorylated to 8-Cl-ATP by mitochondrial ATP synthase^26^. Therefore, AK2 may supply 2-oxo-ADP as a substrate for 2-oxo-ATP synthesis, because AK2 among the adenylate kinase isozymes, is thought to supply ADP as a substrate in ATP synthesis in the mitochondrial intermembrane space^27–29^. The contribution of AK2 to both 2-oxo-Ado-induced cytotoxicity and intracellular accumulation of 2-oxo-ATP, as shown in Fig. 2c,e, supports this interpretation.

Significant switching of intracellular ATP for 2-oxo-ATP is considered to be responsible for 2-oxo-Ado-induced cytotoxicity. However, the mechanism of this process is still unclear, and it is essential to examine whether 2-oxo-ATP itself is cytotoxic. If 2-oxo-ATP itself is not cytotoxic, the depletion of ATP resulting from the process of generating 2-oxo-ATP is likely to be responsible for the cytotoxicity. If this is the case, it also indicates that 2-oxo-ATP cannot substitute for ATP. While a large amount of 2-oxo-ATP is generated through ATP-dependent reactions, such as phosphorylation by ADK and AK2, an equivalent amount of ATP must be consumed, which may be one of the main causes for the depletion of intracellular ATP levels. However, the reduction of intracellular ATP levels after exposure to 2-Cl-Ado is caused by not only consumption of ATP itself but also deamination of AMP^6^, and 8-Cl-ATP may inhibit mitochondrial ATP synthase via binding to the α_tp_β_tp_ site of the enzyme^26^. It has been reported that 2-oxo-ATP reduces the amount of RNA synthesized *in vitro* and increases base substitutions^30^, suggesting that 2-oxo-ATP decreases transcription levels with increased production of abnormal proteins *in vivo*. In the present study, we demonstrated that exposure of T5v cells to 2-oxo-Ado significantly increased the 2-oxo-Ado level in cellular RNA, which was efficiently suppressed by overexpression of hMTH1 (Fig. 2e). Taken together, it is still possible that 2-oxo-ATP itself exerts some cytotoxicity, including disruption of RNA synthesis, mRNA translation, and energy supply.

To evaluate the influence of 2-oxo-ATP itself, we analysed the effects of hMTH1 on changes to intracellular nucleotides caused by 2-oxo-Ado, because hMTH1 hydrolyses 2-oxo-ATP, but does not prevent phosphorylation of 2-oxo-Ado to 2-oxo-ATP. We found that overexpression of hMTH1 in T5MTH1 cells significantly increased intracellular ATP levels and significantly reduced intracellular levels of 2-oxo-ATP as well as 2-oxo-Ado levels in cellular RNA (Fig. 3b,c). This result indicates that 2-oxo-ATP itself contributes to the reduction of intracellular ATP levels. However, there is the possibility that hMTH1 may increase intracellular ATP levels even if 2-oxo-ATP does not contribute to ATP depletion, because in the presence of pyrophosphates, which are produced during hydrolysis of 2-oxo-ATP by hMTH1, ATP is regenerated from ADP-ribose by NUDIX5^31^.

Recently, MTH1 inhibitors have been developed as anti-tumour reagents, because cancer cells express increased levels of MTH1 to survive the increased oxidative conditions that are prevalent in cancer^32, 33^. However, knockdown of *MTH1* or inhibition of MTH1 in cancer cells is not sufficient to introduce cancer cell death^34^. The toxicity of 2-oxo-Ado is not specific to tumour cells as shown in the present study. However, mice can tolerate intraperitoneal administration of 96 mg 2-oxo-Ado per kg per day^5^. We also showed that normal human fibroblasts are resistant to 2-oxo-Ado up to 120 µM, and that a tumour-derived cell line, MOLT4, is highly sensitive to 2-oxo-Ado resulting in cell death at doses of 80–120 µM, while U2OS cells exhibited only growth arrest, suggesting increased expression of MTH1. Here, we propose that a combination of 2-oxo-Ado and an MTH1 inhibitor may have synergistic anti-tumour effects. For such a combination therapy, a cancer-directed drug delivery system would be very important to suppress side effects.

Another possible mechanism of 2-oxo-Ado-induced cytotoxicity is via the p38 MAPK pathway. It has been reported that the p38 MAPK pathway is involved in cell death caused by 8-Cl-cAMP or 8-Cl-Ado^9^. In the present study, we showed that 2-oxo-Ado activates the p38 MAPK pathway through activation of MKK3/MKK6. However, we found that the p38 pathway is dispensable for 2-oxo-Ado-induced cytotoxicity which was demonstrated using inhibitors or siRNAs targeting components of p38 MAPK signalling cascades. Intriguingly, we found that the cytotoxicity caused by 2-oxo-Ado was efficiently suppressed by SB203580, which has been used as a specific inhibitor for p38 MAPKs through activation of MKK3/MKK6, suggesting that SB203580 may exert suppression of cytotoxicity by inhibiting another enzyme. Indeed, we demonstrated that SB203580 inhibits ADK activity *in vitro* using recombinant human ADK protein, while other p38 MAPK inhibitors had little effect on ADK.

SB203580 can effectively, but not fully, inhibit p38 MAPKs at concentrations lower than 1 µM and is often used at 10 to 30 µM to achieve efficient suppression^21^. SB203580 also influences cellular functions through mechanisms other than inhibition of p38 MAPK at 10 µM^35, 36^. Taken together, these data and our present results suggest that there may be erroneous conclusions in publications in which SB203580 was employed to examine the involvement of p38 MAPKs. In particular, results using high concentrations of SB203580 or *in vivo* experiments in which serum concentrations of SB203580 were not measured should be interpreted carefully. Our finding that ADK is a novel target of SB203580 is helpful for the re-interpretation of the results obtained using SB203580, especially when observations are not consistent with results following suppression of p38 MAPK inhibition.

## Methods

### Cells

All cells used in this study are listed in the Supplementary Methods.

### Reagents

All reagents including antibodies and primers used in this study are listed in the Supplementary Methods.

### Trypan blue exclusion assay

A total of 3 × 10^5^ T9 cells in 2 ml of culture medium were added to each well of 6-well plates and incubated for 12–16 h. When Z-VAD was used, cells were incubated for 1 h with Z-VAD or the vehicle before 2-oxo-Ado treatment. The medium was then exchanged with 1 ml of fresh medium without antibiotics, and the cells were treated with the indicated reagents (nucleosides and inhibitors) for various times (0–24 h). When only live cells were counted, cells were washed with phosphate buffered saline (PBS) and then incubated with 200 µl PBS containing 0.0625% trypsin/0.005% EDTA (0.25 × trypsin/EDTA solution) for 5 min. Cell suspensions were mixed with 200 µl of fresh medium. When both live and dead cells were counted, cells harvested using the 0.25 × trypsin/EDTA solution were combined with the removed medium and PBS wash, which may contain floating dead cells, and then centrifuged. Cell pellets were resuspended in 200 µl of fresh medium. Then, 10 µl of the cell suspension was mixed with 10 µl of trypan blue solution (Sigma-Aldrich Japan). Live cells, which were not stained with trypan blue, and dead cells, which were stained with trypan blue, were counted by an automated cell-counting machine (TC20; BIO-RAD, Hercules, CA, USA).

### WST-8 assay

A total of 4 × 10^3^ cells in 100 µl of culture medium were placed in each well of 96-well plates and incubated for 12–16 h. If needed, cells were treated with siRNA (see Materials and Methods: Gene knockdown) for 32–36 h and then replated as described above and incubated for 12–16 h for a total time of 48 h. The medium was changed to fresh culture medium without antibiotics, and cells were treated with the indicated reagents (nucleosides and inhibitors) for 24 h. Fifty microliters of 0.2 × WST-8 reagent (Cell Counting Kit-8; Dojindo, Kumamoto, Japan) diluted in medium was added to each well, and cells were incubated for 2 h. The optical density of each well was measured at 450 nm (OD450) by a microplate reader (infinite M200 pro, TECAN, Männedorf, Switzerland). The background was determined by the OD450 of wells containing medium and reagents but without cells.

### Gene knockdown

All siRNAs used in this study were purchased from Sigma-Aldrich Japan: negative control (Mission_SIC-001), *Adk* (SASI_Mm01_00192948), *Ak2* (SASI_Mm01_00189879), *Mkk3* (SASI_Mm01_00081897) and *Mkk6* (SASI_Mm01_00127382). A total of 8 × 10^3^ T9 cells in 500 µl of culture medium without antibiotics were added to each well of 24-well plates and incubated for 12–16 h. The siRNA solution was prepared at a concentration of 300 nM or 150 nM of each siRNA when two different siRNAs were used simultaneously in Opti-MEM® I Reduced Serum Medium (Thermo Fisher Scientific) containing 1% Lipofectamine® RNAiMAX Reagent (Thermo Fisher Scientific), and incubated for 10 min at room temperature. One hundred microliters of the siRNA solution was added to each well (containing 500 µl of medium) of the 24-well plates. The final concentration of siRNAs was 50 nM.

### RNA extraction and real-time quantitative reverse transcription PCR (qRT-PCR)

Procedures for RNA extraction and qRT-PCR are described in the Supplementary Methods.

### Protein extraction

A total of 2 × 10^5^ T9 cells in 2 ml of culture medium were added to each well of 6-well plates and incubated for 12–16 h. If needed, cells were treated with siRNA (see Materials and Methods: Gene knockdown) for 32–36 h, replated as described above, and incubated for 12–16 h for a total time of 48 h. The medium was then exchanged with 1 ml of fresh medium without antibiotics, and cells were treated with reagents (nucleosides and inhibitors) for various times. The cells were then washed twice with PBS and lysed in 2 × SDS sample buffer (0.125 M Tris-HCl pH 6.8, 4% SDS, 10% glycerol, 2% 2-mercaptoethanol, and 0.005% bromophenol blue) containing 1% phosphatase inhibitor cocktail (Nacalai Tesque, Kyoto, Japan). Lysates were mixed, heated at 95 °C for 5 min, sonicated and then heated again at 95 °C for 5 min. Protein concentrations were determined using an XL-Bradford kit (APRO Science, Tokushima, Japan).

### Western blotting

Western blotting was performed as described in the Supplementary Methods.

### Intracellular nucleotide/nucleoside extraction

A total of 1.4 × 10^6^ cells in 10 ml of culture medium were added to a 10-cm dish and incubated for 12–16 h. The medium was then exchanged with 5 ml of fresh medium without antibiotics, with or without 100 µM 2-oxo-Ado and the indicated inhibitors. The cells were incubated for 6 h, washed twice with ice-cold PBS, and lysed in 250 µl an ice-cold 10% trichloroacetic acid solution (Wako Pure Chemical Industries Ltd, Osaka, Japan) containing 0.2 mM 2′-deoxycoformycin (Santa Cruz Biotechnology, Dallas, TX, USA). Lysates were diluted to about 500 µl with ice-cold PBS, supplemented with 5 µl of 1 M 2,2,6, 6-tetramethylpiperidine 1-oxyl (TEMPO), and placed on ice. Before and after the preparation of cell lysates, the numbers of cells in additional control dishes that received no treatment (two dishes for each) were counted. Lysates were centrifuged at 17,800 × g for 10 min at 4 °C. Three hundred and sixty microliters of each supernatant were mixed with 40 µl of 3 M K_2_HPO_4_ for neutralization and then stored at −80 °C. The volume of the remaining supernatants was measured to calculate the total volume. The concentration of nucleotides and nucleosides in stored solutions was determined by HPLC, and total amounts in original lysates were calculated.

### HPLC

HPLC analysis was performed on an Alliance 2690 HPLC system (Waters, Milford, MA, USA) equipped with a 996 photodiode array (PDA) detector (Waters). A negative ion exchange column (TSKgel DEAE-2SW, Tosoh, Tokyo, Japan, Cat. no. 0007168) was used for separation of nucleotides, and a octadecylsilyl (ODS) column (Handy-ODS, Wako Pure Chemical Industries Ltd, Cat. no. 234–50013) was used for separation of nucleosides. The mobile phases are described in Supplementary Information. The amount of each nucleoside or nucleotide was calculated from the peak area under the curve (AUC) of the PDA absorbance at the wavelength of its maximum absorption: 292 nm for 2-oxo-Ado and its phosphate forms; 259 nm for Ado and its phosphate forms; 263 nm for 2-Cl-Ado and its phosphate forms; 268 nm for AICAR and its phosphate forms. Standard curves for ATP, 2-oxo-ATP and 2-oxo-Ado were used to determine their absolute amounts. 2-Oxo-ATP was partially dephosphorylated by alkaline phosphatase (Roche Applied Science, Penzberg, Germany), and the resulting four HPLC peaks corresponding to 2-oxo-Ado, 2-oxo-AMP, 2-oxo-ADP and 2-oxo-ATP were used to identify peaks of 2-oxo-AMP and 2-oxo-ADP. Quantities of 2-oxo-AMP and 2-oxo-ADP were calculated based on the maximum absorption coefficient of 2-oxo-ATP because these three nucleotides have the same base moiety and are considered to have the same absorption coefficient. Chromatograms of the reaction products of ADK with 2-Cl-Ado or AICAR showed novel peaks with the same absorption spectrums as those of their parent molecule, which were considered to correspond to each monophosphate form.

### LC-MS/MS

LC-MS/MS analysis was performed as described in the Supplementary Methods.

### Enzyme assay

rhADK was purchased from NOVO CIB (Cat. no. E-Nov5, Lyon, France). One unit of enzyme activity was defined as activity converting 1.0 µM of inosine and ATP to IMP and ADP per minute at 37 °C (pH 8). Previously reported reaction conditions were used^37^ with some modifications. When 2-oxo-Ado, 2-Cl-Ado or AICAR was used as a substrate, 100 µM modified Ado was reacted with 1 mM ATP, 295 nU/µl rhADK, and various concentrations of inhibitors in 100 µl of a reaction mixture containing 50 mM Tris-HCl (pH 7.5), 40 mM KCl, 1 mM MgCl_2_, 1 mM dithiothreitol, 0.1 mg/ml bovine serum albumin, and 1% dimethyl sulfoxide (DMSO) for 25 min at 37 °C. Reactions were stopped by adding 60 µl of the incubated reaction mixture to 60 µl of 25 mM EDTA. The products were placed on ice and then subjected to HPLC analysis. The reaction products could be stored at −80 °C before HPLC analysis. When Ado was used as a substrate, the same conditions used for modified Ado were applied with the following modifications. Nucleoside concentrations varied from 100 µM modified Ado to 0.5 µM Ado because Ado inhibits ADK activity at higher concentrations^38^, and the concentration of rhADK varied from 295 to 0.2 nU/µl to obtain a linear reaction within 25 min. The background was determined by analysing samples containing only substrates without rhADK or inhibitors.

### Statistical analysis

Statistical analyses were conducted using JMP 11.00 (SAS Institute, Cary, NC, USA). To assess statistical significance, we performed one-way or two-way ANOVA. Results obtained by standard least square fits are shown. For multiple comparison tests, ANOVA was followed by a *post hoc* Tukey’s honestly significant difference (HSD) test. A value of p < 0.05 was considered as statistically significant (*p < 0.05, **p < 0.01, ***p < 0.001, and ****p < 0.0001).

## Electronic supplementary material

Supplementary Information

## References

[1] Cherbuliez E, Bernhard K (1932). Recherches sur la graine de croton. I. Sur le crotonoside (2-oxy-6-amino-purine-d-riboside). Helvetica Chimica Acta.

[2] Fuhrman FA, Fuhrman GJ, Nachman RJ, Mosher HS (1981). Isoguanosine: isolation from an animal. Science.

[3] Ewing PL, Schlenk F, Emerson GA (1949). Comparison of smooth muscle effects of crotonoside (isoguanosine) and adenosine. J Pharmacol Exp Ther.

[4] Huang M, Daly JW (1972). Accumulation of cyclic adenosine monophosphate in incubated slices of brain tissue. 1. Structure-activity relation of agonists and antagonists of biogenic amines and of tricyclic tranquilizers and antidepressants. J Med Chem.

[5] Kim JH, Lee SJ, Han YB, Moon JJ, Kim JB (1994). Isolation of isoguanosine from Croton tiglium and its antitumor activity. Arch Pharm Res.

[6] Bastin-Coyette L (2008). Mechanisms of cell death induced by 2-chloroadenosine in leukemic B-cells. Biochem Pharmacol.

[7] Koshiba M (2002). 2-Chloroadenosine but not adenosine induces apoptosis in rheumatoid fibroblasts independently of cell surface adenosine receptor signalling. Br J Pharmacol.

[8] Gandhi V (2001). 8-chloro-cAMP and 8-chloro-adenosine act by the same mechanism in multiple myeloma cells. Cancer Res.

[9] Ahn YH, Jung JM, Hong SH (2005). 8-Chloro-cyclic AMP-induced growth inhibition and apoptosis is mediated by p38 mitogen-activated protein kinase activation in HL60 cells. Cancer Res.

[10] Crawley, J. B. *et al*. T cell proliferation in response to interleukins 2 and 7 requires p38MAP kinase activation. *J Biol Chem* **272**, 15023–15027, doi:10.1074/jbc.272.23.15023 (1997). 10.1074/jbc.272.23.15023 9169478

[11] Schwenger P (1997). Sodium salicylate induces apoptosis via p38 mitogen-activated protein kinase but inhibits tumor necrosis factor-induced c-Jun N-terminal kinase/stress-activated protein kinase activation. Proc Natl Acad Sci USA.

[12] Fujikawa K, Kamiya H, Yakushiji H, Nakabeppu Y, Kasai H (2001). Human MTH1 protein hydrolyzes the oxidized ribonucleotide, 2-hydroxy-ATP. Nucleic Acids Res.

[13] Callus BA, Vaux DL (2007). Caspase inhibitors: viral, cellular and chemical. Cell Death Differ.

[14] Tait SW, Green DR (2010). Mitochondria and cell death: outer membrane permeabilization and beyond. Nat Rev Mol Cell Biol.

[15] Ugarkar, B. G. *et al*. Adenosine kinase inhibitors. 1. Synthesis, enzyme inhibition, and antiseizure activity of 5-iodotubercidin analogues. *J Med Chem* **43**, 2883–2893, doi:10.1021/jm000024g (2000). 10.1021/jm000024g 10956196

[16] Barkulis SS, Lehninger AL (1951). Myokinase and the adenine nucleotide specificity in oxidative phosphorylations. J Biol Chem.

[17] Yoshimura D (2003). An oxidized purine nucleoside triphosphatase, MTH1, suppresses cell death caused by oxidative stress. J Biol Chem.

[18] Derijard, B. *et al*. Independent human MAP-kinase signal transduction pathways defined by MEK and MKK isoforms. *Science* **267**, 682–685, doi:10.1126/science.7839144 (1995). 10.1126/science.7839144 7839144

[19] Raingeaud J, Whitmarsh AJ, Barrett T, Derijard B, Davis RJ (1996). MKK3- and MKK6-regulated gene expression is mediated by the p38 mitogen-activated protein kinase signal transduction pathway. Molecular and Cellular Biology.

[20] Rouse J (1994). A Novel Kinase Cascade Triggered by Stress and Heat-Shock That Stimulates Mapkap Kinase-2 and Phosphorylation of the Small Heat-Shock Proteins. Cell.

[21] Cuenda, A. *et al*. SB203580 is a specific inhibitor of a MAP kinase homologue which is stimulated by cellular stresses and interleukin-1. *Febs Letters* **364**, 229–233, doi:10.1016/0014-5793(95)00357-F (1995). 10.1016/0014-5793(95)00357-f 7750577

[22] Ambrosino C (2003). Negative Feedback Regulation of MKK6 mRNA Stability by p38 Mitogen-Activated Protein Kinase. Molecular and Cellular Biology.

[23] Corton JM, Gillespie JG, Hawley SA, Hardie DG (1995). 5-aminoimidazole-4-carboxamide ribonucleoside. A specific method for activating AMP-activated protein kinase in intact cells?. Eur J Biochem.

[24] Marone G, Plaut M, Lichtenstein LM (1978). Characterization of a specific adenosine receptor on human lymphocytes. J Immunol.

[25] Marone G, Findlay SR, Lichtenstein LM (1979). Adenosine receptor on human basophils: modulation of histamine release. J Immunol.

[26] Chen LS (2009). Inhibition of ATP synthase by chlorinated adenosine analogue. Biochem Pharmacol.

[27] Noma, T. Dynamics of nucleotide metabolism as a supporter of life phenomena. *J Med Invest* **52**, 127–136, doi:10.2152/jmi.52.127 (2005). 10.2152/jmi.52.127 16167529

[28] Nobumoto M, Yamada M, Song S, Inouye S, Nakazawa A (1998). Mechanism of mitochondrial import of adenylate kinase isozymes. J Biochem.

[29] Dzeja P, Terzic A (2009). Adenylate kinase and AMP signaling networks: metabolic monitoring, signal communication and body energy sensing. Int J Mol Sci.

[30] Kamiya H, Suzuki A, Kawai K, Kasai H, Harashima H (2007). Effects of 8-hydroxy-GTP and 2-hydroxy-ATP on *in vitro* transcription. Free Radic Biol Med.

[31] Wright RHG (2016). ADP-ribose-derived nuclear ATP synthesis by NUDIX5 is required for chromatin remodeling. Science.

[32] Huber KV (2014). Stereospecific targeting of MTH1 by (S)-crizotinib as an anticancer strategy. Nature.

[33] Gad H (2014). MTH1 inhibition eradicates cancer by preventing sanitation of the dNTP pool. Nature.

[34] Kettle JG (2016). Potent and Selective Inhibitors of MTH1 Probe Its Role in Cancer Cell Survival. J Med Chem.

[35] Lali FV, Hunt AE, Turner SJ, Foxwell BM (2000). The pyridinyl imidazole inhibitor SB203580 blocks phosphoinositide-dependent protein kinase activity, protein kinase B phosphorylation, and retinoblastoma hyperphosphorylation in interleukin-2-stimulated T cells independently of p38 mitogen-activated protein kinase. J Biol Chem.

[36] Zhang H (2012). Induction of autophagy in hepatocellular carcinoma cells by SB203580 requires activation of AMPK and DAPK but not p38 MAPK. Apoptosis.

[37] Long MC, Parker WB (2006). Structure-activity relationship for nucleoside analogs as inhibitors or substrates of adenosine kinase from Mycobacterium tuberculosis. I. Modifications to the adenine moiety. Biochem Pharmacol.

[38] Fox IH, Palella TD, Thompson D, Herring C (1982). Adenosine metabolism: modification by S-adenosylhomocysteine and 5′-methylthioadenosine. Arch Biochem Biophys.

