# Supplementary material for: 2-Oxoadenosine induces cytotoxicity through intracellular accumulation of 2-oxo-ATP and depletion of ATP but not via the p38 MAPK pathway
Source: Sci Rep. 2017 Jul 26;7:6528. doi: 10.1038/s41598-017-06636-8 (PMC5529524; doi:10.1038/s41598-017-06636-8)
Supplement: Supplementary file 1 — Supplementary Information [file 41598_2017_6636_MOESM1_ESM.docx]

*Supplementary Information for:*

**2-Oxoadenosine induces cytotoxicity through intracellular accumulation of 2-oxo-ATP and depletion of ATP but not via the p38 MAPK pathway**

Shinji Asada^1,2^, Eiko Ohta^1^, Yoriko Akimoto^1^, Nona Abolhassani^1^, Daisuke Tsuchimoto^1^, Yusaku Nakabeppu^1,^*

^1^ Division of Neurofunctional Genomics, Department of Immunobiology and Neuroscience, Medical Institute of Bioregulation, Kyushu University, Fukuoka 812-8582, Japan

^2^ Department of Medicine and Clinical Science, Graduate School of Medical Sciences, Kyushu University, Fukuoka 812-8582, Japan

***** Correspondence and requests for materials should be addressed to Y.N. (email: [yusaku@bioreg.kyushu-u.ac.jp](mailto:yusaku@bioreg.kyushu-u.ac.jp))

This file contains:

Supplementary Figures S1–S9

Supplementary Methods

Supplementary References

**Supplementary Figure S1. 2-Oxo-Ado is cytotoxic in human cell lines.** Numbers of live and dead cells (a, b: WI38; c, d: U2OS; e, f: MOLT4) were determined by trypan blue exclusion at 24 h after incubation in the presence of various concentrations of 2-oxo-Ado. (a, c, e) Relative ratio of the total number of cells (open box, live cells; gray box, dead cells) to the initial number of total cells is shown. (b, d, f) Survival rate is shown as a percentage of live cells at each concentration of 2-oxo-Ado. Data are the mean ± SD of three experiments.

**Supplementary Figure S2. Efficiency of siRNA-mediated knockdown of *Adk* and *Ak2* expression.** (a) Gene expression levels in T9 cells were determined by qRT-PCR at 48 h after treatment with siRNAs. Results were normalized against *Gapdh* expression. Values (fold changes) of gene expression levels relative to samples without siRNA treatment (none) are shown as the mean ± SD of three experiments. NC, negative control siRNA.

**Supplementary Figure S3. ADK inhibitor and siRNAs against *Adk* and *Ak2* do not alter the intracellular concentration of 2-oxo-Ado.** Intracellular 2-oxo-Ado concentrations of T9 cells were determined by HPLC at 6 h after incubation in the presence or absence of 100 µM 2-oxo-Ado. (a) Cells were simultaneously treated with the vehicle (0.3% DMSO) or 0.1 µM Itu in the presence of 2-oxo-Ado. (b) Cells were subjected to *Adk* and *Ak2* knockdown (KD) prior to 2-oxo-Ado treatment. Results are shown as the mean ± SD of three experiments. NC, negative control siRNA; ND, not detected. Results were statistically analysed by one-way ANOVA and *post-hoc* Tukey’s HSD test. ns, not significant; **p* < 0.05; ***p* < 0.01; *****p* < 0.001.

**Supplementary Figure S4. All used inhibitors block activity of the p38 MAPK pathway after 2-oxo-Ado treatment.** (a) Protein levels of T9 cells were determined by western blot analysis after incubation for 9 h in the presence of 50 µM 2-oxo-Ado with or without the indicated inhibitors. SB203580, SB202190, VX745 and BIRB796 (p38 MAPK inhibitors), 10 µM. Itu, 0.1 µM. Vehicle control, 0.1% DMSO. Ten micrograms of total protein were applied to each lane. (b) Quantification of protein levels in a. Values (fold change) of phosphorylation levels relative to that at time 0 are shown as the mean of two experiments.


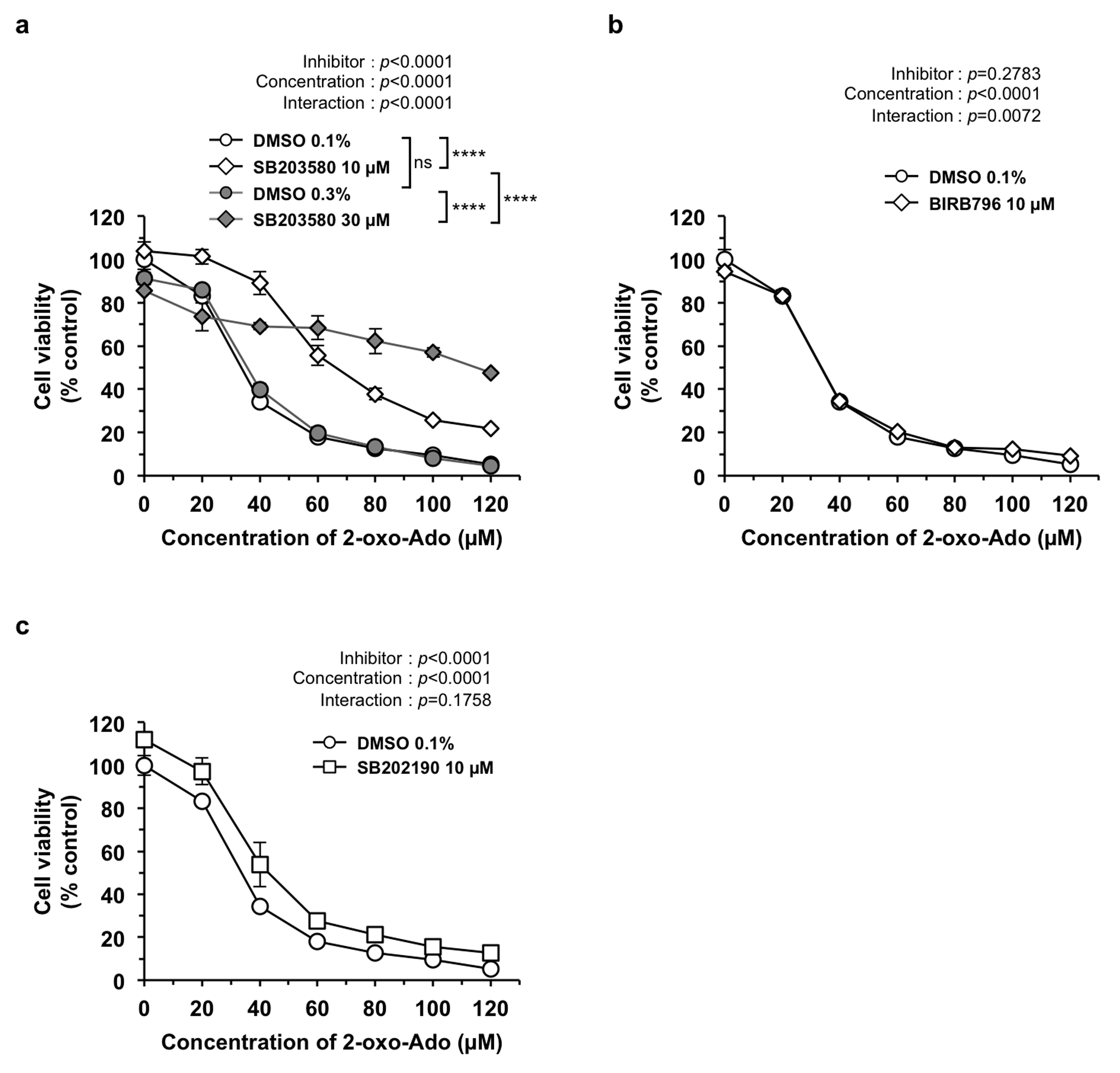


**Supplementary Figure S5.** **SB203580 dose-dependently blocks 2-oxo-Ado-induced cell death.** (a–c) Viability of T9 cells was determined by WST-8 assays after incubation for 24 h in the presence of various concentrations of 2-oxo-Ado with or without the indicated inhibitors. Vehicle control of 10 µM SB203580 (a), BIRB796 (b) and SB202190(c), 0.1% DMSO. Vehicle control of 30 µM SB203580 (a), 0.3% DMSO. Values (% control) of cell viability relative to that of each vehicle control in the absence of 2-oxo-Ado are shown as the mean ± SD of three experiments. Results were statistically analysed by two-way ANOVA (a–c) and *post-hoc* Tukey’s HSD test (a). *****p* < 0.0001.

**Supplementary Figure S6.** **Gene knockdown of *MKK3* and *MKK6* blocks activation of p38 MAPK pathway.** (a) Gene-expression levels in T9 cells were determined by qRT-PCR 48 h after treatment with siRNAs. Results were normalized against *Gapdh* expression. Values (fold change) of gene-expression levels relative to that of NC siRNA-samples are shown as the mean ± SD of three experiments. (b) Protein levels in T9 cells were determined by western blot analysis. Cells were treated with target siRNA or NC siRNA for 48 h and then incubated in the presence of various concentrations of 2-oxo-Ado for 9 h. Eight micrograms of total protein were applied to each lane. p-; phosphorylated. (c, d) Quantification of protein levels in b. Values (fold change) of phosphorylation levels relative to that of NC siRNA samples in the absence of 2-oxo-Ado are shown as the mean of two experiments.

**Supplementary Figure S7. SB203580 blocks AMPK phosphorylation by AICAR, an Adk-dependent reaction.** (a) Protein levels of T9 cells were determined by western blot analysis after incubation for 1 h in the presence or absence of 0.5 mM AICAR and the indicated inhibitors. SB203580, SB202190, VX745 and BIRB796, 10 µM. Itu, 0.1 µM. Vehicle control, 0.77% DMSO. Eight micrograms of total protein were applied to each lane. (b) Quantification of total and phosphorylated AMPK levels in A. Values (fold change) of phosphorylation levels relatives to that of control in the absence of AICR are shown as the mean ± SD of three experiments. Results were statistically analysed by two-way ANOVA and *post-hoc* Tukey’s HSD test.

**
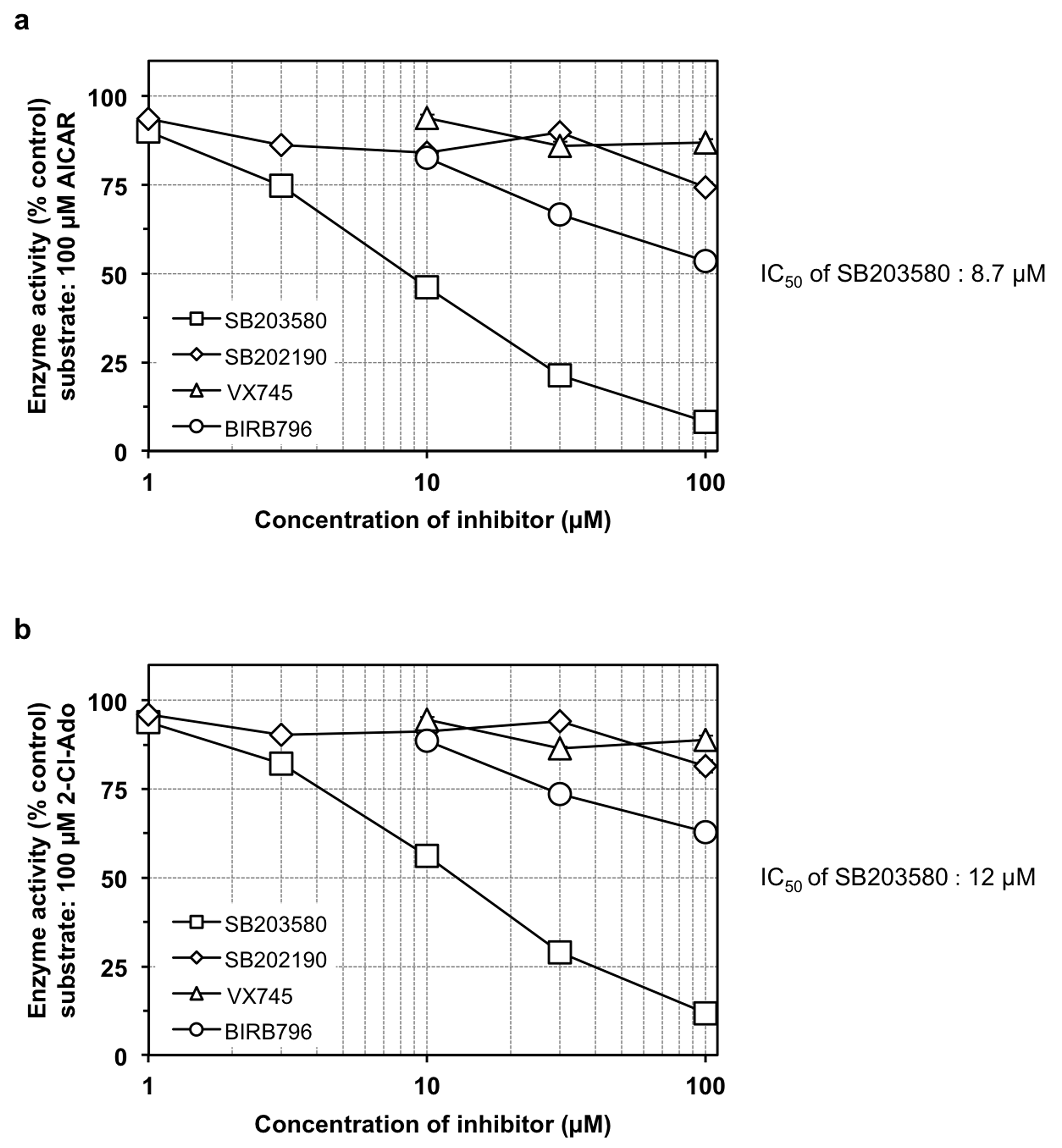
**

**Supplementary Figure S8. SB203580 directly inhibits adenosine kinase activity for adenosine analogue substrates.** (a, b) Human recombinant ADK, ATP and nucleosides. AICAR (a) or 2-Cl-Ado (b) were reacted in the presence of various concentrations of the indicated inhibitors. Enzyme activity was determined by the amount of monophosphate products, ZMP (A) or 2-Cl-AMP (b). The amount of product was determined by HPLC. Values (% control) of enzyme activity relative to that in the absence of inhibitors are shown as the mean ± SD of three experiments for each kind of substrate. IC50 was calculated graphically.

**
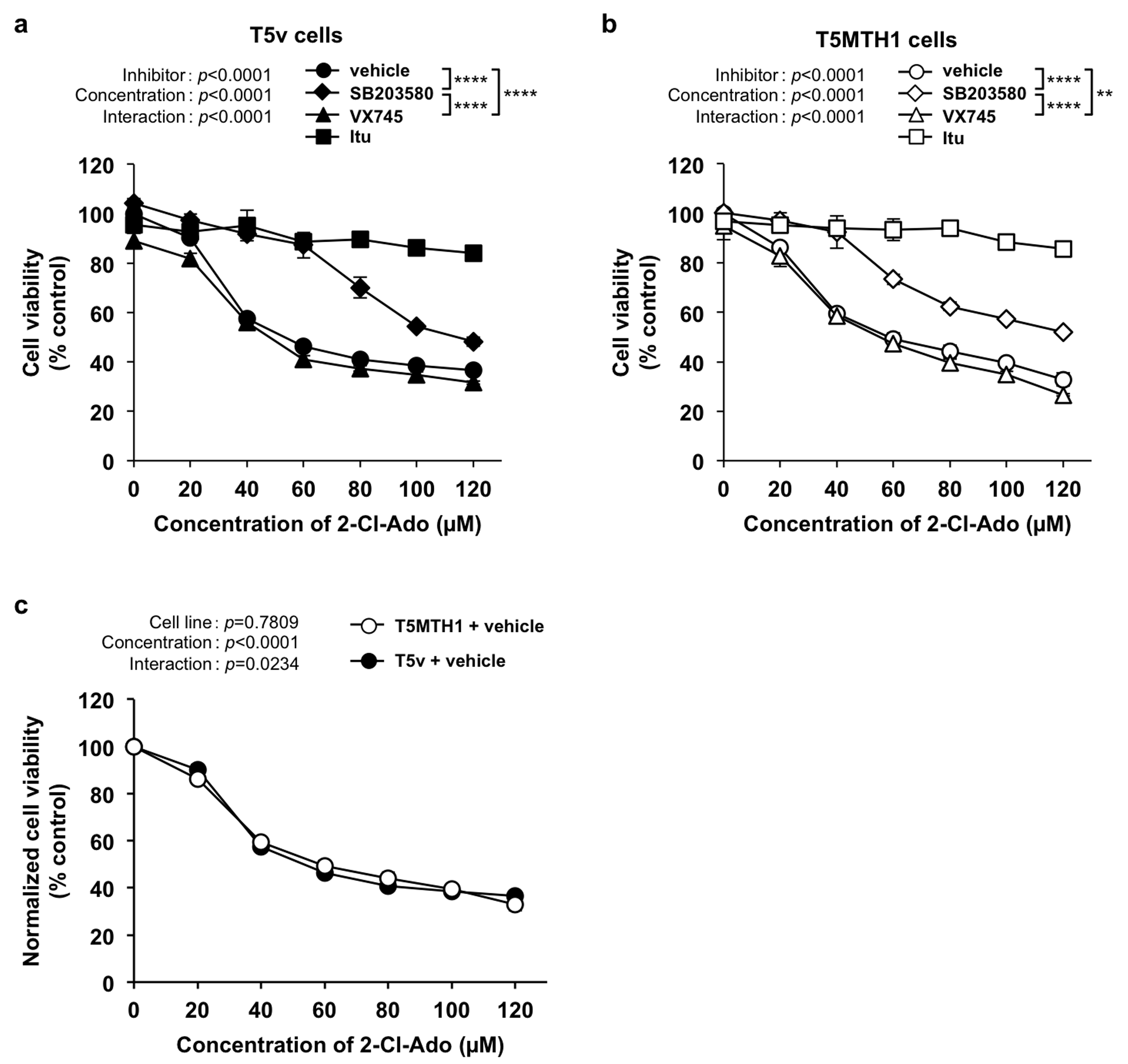
**

**Supplementary Figure S9. SB203580 blocks 2-Cl-Ado-induced cell death, an ADK-dependent reaction, but not via inhibition of the p38 MAPK pathway.** (a–c) Viability of T5v and T5MTH1 cells was determined by WST-8 assays after incubation for 24 h in the presence of various concentrations of 2-Cl-Ado with or without the indicated inhibitors. SB203580 and VX745, 10 µM. Itu, 0.1 µM. Vehicle control, 0.1% DMSO. Values (% control) of cell viability relative to that of the vehicle control in the absence of 2-Cl-Ado are shown as the mean ± SD of three experiments for each cell line. ***p* < 0.01; *****p* < 0.0001. Itu had a significant difference (*p* < 0.0001) compared with the other two inhibitors or vehicle control in T5v and T5MTH1 cells.

**Supplemental Methods**

**Cells.** We previously established the following MEF lines: wild-type MEF (T9), MTH1-null MEF (T5), T5MTH1; T5 cells transfected with recombinant human MTH1-expressing plasmid, and T5v; T5 cells transfected with an empty vector ^1^. These cells were maintained as described previously ^1^. WI38 cells, normal diploid human fibroblasts derived from lung tissue, and MOLT4 cells, a human acute lymphoblastic leukaemia cell line, were obtained from the Japanese Collection of Research Bioresources Cell Bank. U2OS cells, a human osteosarcoma cell line were kindly provided by Dr. Thomas Helleday (Karolinska Institutet, Stockholm, Sweden).

**Reagents.** SB203580 was purchased from Invivogen (San Diego, CA, USA). SB202190, VX745, BIRB796 and 2’-deoxycoformycin were from Santa Cruz Biotechnology (Dallas, TX, USA). 5-Iodotubercidin (Itu), adenosine (Ado), adenosine monophosphate (AMP), adenosine triphosphate (ATP), 2-chloroadenosine (2-Cl-Ado) and 2,2,6,6-tetramethylpiperidine 1-oxyl (TEMPO) were from Sigma-Aldrich Japan (Tokyo, Japan). Adenosine diphosphate (ADP) was from Yamasa (Tokyo, Japan). 2-Oxoadenosine (2-oxo-Ado) and 2-oxoadenosine triphosphate (2-oxo-ATP) were from Jena Bioscience (supplied as isoguanosine and 2-hydroxy-ATP, Thüringen, Germany). 5-Aminoimidazole-4-carboxamide-1-β-D-ribofuranoside (AICAR) was from Tokyo Chemical Industry (Tokyo, Japan). Z-VAD(OMe)-FMK (Z-VAD) and Ponceau S were from MP Biomedicals (Santa Ana, CA, USA), Hoechst 33342 was from Calbiochem (San Diego, CA, USA). Propidium iodide (PI) was from Sigma-Aldrich Japan.

**Antibodies.** Antibodies (Abs) used in this study were a rabbit anti-AMPKα Ab (Cell Signaling Technology Japan [CST Japan], Tokyo, Japan, cat. no. 2532, used at 1:1000), rabbit monoclonal anti-phospho-AMPKα (Thr172) (40H9) Ab (CST Japan, cat. no. 2535, 1:1000), rabbit anti-phospho-MKK3 (Ser189)/MKK6 (Ser207) Ab (CST Japan, cat. no. 9231, 1:1000), rabbit anti-p38 MAPK Ab (CST Japan, cat. no. 9212, 1:1000), rabbit anti-phospho-p38 MAPK (Thr180/Tyr182) Ab (CST Japan, cat. no. 9211, 1:1000), rabbit anti-MAPKAPK-2 Ab (CST Japan, cat. no. 3042, 1:1000), rabbit monoclonal anti-phospho-MAPKAPK-2 (Thr334) (27B7) Ab (CST Japan, cat. no. 9211, 1:1000), mouse anti-β-actin Ab (Sigma-Aldrich Japan, cat. no. A5316, 1:6000), rabbit monoclonal anti-caspase-3 (8G10) Ab (CST Japan, cat. no. 9665, 1:1000), mouse monoclonal anti-caspase-9 (C9) Ab (CST Japan, cat. no. 9508, 1:1000), goat anti-rabbit IgG Ab conjugated to horseradish peroxidase (HRP) (CST Japan, cat. no. 7074, 1:2000) and horse anti-mouse IgG conjugated to HRP (CST Japan, cat. no. 7076, 1:3000).

**Primers.** Quantitative real-time PCRs were performed with the following primers sets: *Adk* (5′-AGCGCTGAGTGAAAATGTGC-3′, 5′-AATTCTGCGTAGAGCCACCA-3′), *Ak2* (5′-GGAGATTCCGAAGGGCATCC-3′, 5′-ACCATGGCTCTCAGCATGTC-3′), *Mkk3* (5′-TACATGGCCCCTGAGAGGAT-3′, 5′-GGTGTGCCCCAAGACTCATA-3′), *Mkk6* (5′-AGTCGAAAGGCAAGAAGCGA-3′, 5′-GAGTCTAAATCCCGAGGCGG-3′) and *Gapdh* (5′-AAATGGTGAAGGTCGGTGTG-3′, 5′-TGAAGGGGTCGTTGATGG-3′).

**Western blotting.** Protein extracts were separated on 12.5% SDS-polyacrylamide gels and then transferred onto polyvinylidene fluoride membranes (Immobilon®-P; Merck Millipore, Billerica, MA, USA). The membranes were washed with TBST (10 mM Tris-HCl pH 7.5, 150 mM NaCl, and 0.1% Tween 20) and blocked in TBST containing 5% dry non-fat milk for 1 h at room temperature. Membranes were incubated in TBST containing a primary antibody or 5% non-fat milk containing anti-caspase-3 or anti-caspase-9 antibody for 16 h at 4°C. Then, the membranes were washed with TBST and incubated in TBST containing an appropriate secondary antibody for 1 h at room temperature. After washing with TBST, bound antibodies were detected with EzWestLumi plus (ATTO, Tokyo, Japan), and digitized images were obtained using an Ez-Capture MG (ATTO).

**RNA extraction and real-time quantitative reverse transcription-PCR (qRT-PCR)**. Total RNAs were extracted from cells in 24-well plates using ISOGEN (Nippon Gene, Tokyo, Japan) according to the manufacturer’s instructions. cDNAs were synthesized using a High Capacity cDNA Reverse Transcription Kit (Life Technologies Japan, Tokyo, Japan). Quantitative real-time PCR was performed with 10 ng cDNA, 200 nM of primers and Thunderbird® SYBR® qPCR Mix (Toyobo, Osaka, Japan) using a Thermal Cycler Dice® Real Time System Single (Takara Bio, Shiga, Japan). The ΔΔCT method was used to calculate values relative to *Gapdh* mRNA as an internal control.

**Preparation of stable-isotope labelled 2-oxoadenosine.** [^13^C, ^15^N]-labelled ATP (10 mM, Silantes GmbH, Munich, Germany) was incubated in the presence of 40 mM Fe(II)-EDTA/ 50 mM Tris-HCl (pH 8.5) for 30 min at 37°C with vigorous shaking, in the dark as described previously ^2^. The reaction was terminated by adding 20 mM TEMPO and then placed on ice for 3 min. [^13^C, ^15^N]-labelled 2-oxo-ATP was purified by three-step HPLC: 1st anion-exchange HPLC on a MiniQ column (GE Healthcare Japan, Tokyo, Japan), a reverse-phase HPLC on a SunFire C18 column (Waters), and 2nd anion-exchange HPLC on a RESOURCE Q column (GE Healthcare). The purified [^13^C, ^15^N]-labelled 2-oxo-ATP was converted to the nucleoside (2-oxo-Ado) using alkaline phosphatase from calf intestine (Roche Applied Science, Penzberg, Germany), and the [^13^C, ^15^N]-labelled 2-oxo-Ado was purified by a reverse-phase HPLC on a Wakopak Handy ODS column (Wako Pure Chemical Industries Ltd, Osaka, Japan). Molecular mass (299.1) and spectroscopic and chromatographic behaviours of the [^13^C, ^15^N]-labelled 2-oxo-Ado were confirmed in comparison with those of non-isotopic [^12^C, ^14^N]-2-oxo-Ado obtained from Jena Bioscience (Thüringen, Germany).

**Liquid chromatography-tandem mass spectrometry (LC-MS/MS).** 2-Oxo-Ado levels in RNA were determined as follows. Total RNAs were extracted from 2.0 × 10^5^ cells in 6-well plates using ISOGEN. RNA samples were digested with 5 µg/ml RNase A (Sigma-Aldrich Japan), 0.04 U/µl Nuclease P1 (Wako Pure Chemical Industries Ltd), 0.34 U/µl alkaline phosphatase (Sigma Aldrich Japan), and 0.5 U/ml phosphodiesterase I (US Biological, Salem, MA, USA) in the presence of 10 mM TEMPO, 100 µM 2’-deoxycoformycin and 100 µg/ml tetrahydrouridine (Calbiochem, Merck Millipore). To allow for accurate quantitation of 2-oxo-Ado and account for the signal variability of the mass spectrometer, [^13^C, ^15^N]-labelled 2-oxo-Ado was added as a stable-isotope labelled internal standard to all samples.

LC-MS/MS analysis of 2-oxo-Ado in the digested RNA samples was performed using a Nexera X2 LC system (Shimadzu, Kyoto, Japan) connected to a triple-quadrupole mass spectrometer API3200 (AB SCIEX, Framingham, MA, USA). The digested RNA samples were applied to an Acclaim^TM^ PolarAdvantage column (3 µm, 3.0 mm × 250 mm, Thermo Fisher Scientific Inc., Sunnyvale, CA, USA) maintained at 24°C, and eluted at a flow rate 0.2 ml/min using a mobile phase buffer (0.1% acetic acid). The eluent was monitored at 254 nm using an SPD-20A UV/Vis detector (Shimadzu). The amounts of guanosine and adenosine were calculated based on the absorbance of known amounts of standard nucleosides.

The mass spectrometric analysis was carried out in the positive ionization mode with a turbo ion spray source using nitrogen gas as a nebulizer and curtain gas. Mass spectrometer parameters optimised for 2-oxo-Ado were as follows: curtain gas (CUR) 40 psi; collision gas (CAD): 4; ion spray voltage (IS): 5500 V; temperature 700°C; ion source gas 1 (GS1): 80 psi; ion source gas 2 (GS2): 50 psi; declustering potential (DP): 36 V; entrance potential (EP): 3 V; collision energy (CE): 35 V; collision cell exit potential (CXP): 3 V. Data were obtained in multiple reaction monitoring (MRM) mode, using transitions of m/z 284.2 to 152.2 for 2-oxo-Ado and m/z 299.1 to 162.2 for [^13^C, ^15^N]-labelled 2-oxo-Ado. Data acquisition and quantification were performed by Analyst 1.6.2 software (AB SCIEX).

**Supplementary References**

1. Yoshimura, D. *et al.* An oxidized purine nucleoside triphosphatase, MTH1, suppresses cell death caused by oxidative stress. *J Biol Chem* **278**, 37965-37973, doi:10.1074/jbc.M306201200 (2003).
2. Fujikawa, K. *et al.* Human MTH1 protein hydrolyzes the oxidized ribonucleotide, 2-hydroxy-ATP. *Nucleic Acids Res* **29**, 449-454, doi:10.1093/nar/29.2.449 (2001)
